# A *Plasmodium falciparum *FcB1-schizont-EST collection providing clues to schizont specific gene structure and polymorphism

**DOI:** 10.1186/1471-2164-10-235

**Published:** 2009-05-19

**Authors:** Isabelle Florent, Betina M Porcel, Elodie Guillaume, Corinne Da Silva, François Artiguenave, Eric Maréchal, Laurent Bréhélin, Olivier Gascuel, Sébastien Charneau, Patrick Wincker, Philippe Grellier

**Affiliations:** 1 FRE3206 CNRS/MNHN, USM504, Biologie Fonctionnelle des Protozoaires, RDDM, Muséum National d'Histoire Naturelle, Paris, France; 2 UMR-CNRS 8030, Genoscope, Evry, France; 3 UMR-CNRS 5168, CEA, INRA, Université Joseph Fourier, Grenoble, France; 4 UMR-CNRS 5506, Laboratoire d'Informatique, de Robotique et de Micro-électronique de Montpellier, Université de Montpellier II, Montpellier, France

## Abstract

**Background:**

The *Plasmodium falciparum *genome (3D7 strain) published in 2002, revealed ~5,400 genes, mostly based on *in silico *predictions. Experimental data is therefore required for structural and functional assessments of *P. falciparum *genes and expression, and polymorphic data are further necessary to exploit genomic information to further qualify therapeutic target candidates. Here, we undertook a large scale analysis of a *P. falciparum *FcB1-schizont-EST library previously constructed by suppression subtractive hybridization (SSH) to study genes expressed during merozoite morphogenesis, with the aim of: 1) obtaining an exhaustive collection of schizont specific ESTs, 2) experimentally validating or correcting *P. falciparum *gene models and 3) pinpointing genes displaying protein polymorphism between the FcB1 and 3D7 strains.

**Results:**

A total of 22,125 clones randomly picked from the SSH library were sequenced, yielding 21,805 usable ESTs that were then clustered on the *P. falciparum *genome. This allowed identification of 243 protein coding genes, including 121 previously annotated as hypothetical. Statistical analysis of GO terms, when available, indicated significant enrichment in genes involved in "entry into host-cells" and "actin cytoskeleton". Although most ESTs do not span full-length gene reading frames, detailed sequence comparison of FcB1-ESTs versus 3D7 genomic sequences allowed the confirmation of exon/intron boundaries in 29 genes, the detection of new boundaries in 14 genes and identification of protein polymorphism for 21 genes. In addition, a large number of non-protein coding ESTs were identified, mainly matching with the two A-type rRNA units (on chromosomes 5 and 7) and to a lower extent, two atypical rRNA loci (on chromosomes 1 and 8), TARE subtelomeric regions (several chromosomes) and the recently described telomerase RNA gene (chromosome 9).

**Conclusion:**

This FcB1-schizont-EST analysis confirmed the actual expression of 243 protein coding genes, allowing the correction of structural annotations for a quarter of these sequences. In addition, this analysis demonstrated the actual transcription of several remarkable non-protein coding loci: 2 atypical rRNA, TARE region and telomerase RNA gene. Together with other collections of *P. falciparum *ESTs, usually generated from mixed parasite stages, this collection of FcB1-schizont-ESTs provides valuable data to gain further insight into the *P. falciparum *gene structure, polymorphism and expression.

## Background

Malaria, the most devastating parasitic human disease, is due to infections by intracellular protozoan parasites belonging to the *Plasmodium *genus transmitted by *Anopheles *mosquitoes [1]. Four *Plasmodium *species are pathogenic to humans, with *P. falciparum *responsible for 90% of all reported cases of malaria, which causes 1.5 to 2.7 million deaths per annum [2]. No efficient vaccine is currently available, despite ongoing efforts over the last decades [3], and alternative drugs and targets are being investigated to fight the drug-resistant parasites that have emerged since the 1960s and are continuously spreading [4].

Deciphering of the *P. falciparum *genome in 2002 [5] revealed 5,300–5,400 genes, 60% of which were initially annotated as hypothetical, since no function could be ascribed to them based on sequence similarity. The PlasmoDB database  gathers genomic and post-genomic data regarding *P. falciparum *and related species, and the last inventory (version 5.4) indicated 5,484 coding genes, 3,155 (~57%) of which were still annotated as hypothetical or hypothetical conserved (i.e. conserved throughout the *Plasmodium *genus). Determining gene structures is particularly difficult in the case of *P. falciparum*, not only because most genes are devoid of characterized orthologs on which gene models could be based, but also because of the very high A-T content of the genome, i.e. 80.6% on average [5]. Gene-coding predictions, based on several algorithms (PHAT, GeneFinder, GlimmerM, Hexamer) have however allowed models to be proposed for *P. falciparum *genes [6], but these gene models require experimental data to be validated.

We previously reported the construction of an EST library using highly synchronized *P. falciparum *parasites of the FcB1 strain (from Colombia) to isolate genes selectively expressed during merozoite morphogenesis [7]. The merozoite is the tiny (1 μm) free form of the parasite that is able to recognize, bind and then invade erythrocytes [8]. This very specialized cell displays a number of remarkable features, including a surface coat composed of highly polymorphic merozoite surface proteins (MSPs), some of which were shown to be essential for parasite invasion and survival [8,9]. The merozoite is also equipped with specialized organelles, such as micronemes, rhoptries and dense granules, devoted to invasion. For example, erythrocyte binding antigens, stored in micronemes, are released prior to invasion and allow host cell recognition, while rhoptry proteins stored in rhoptries are release later and are believed to participate in parasitophorous vacuole formation [8]. Shortly after invasion, internalized merozoites differentiate into rings, thus losing their shape, and specific invasive organelles and rings eventually differentiate into haemoglobin-degrading trophozoites, about 20 h after invasion [10]. Nuclear division takes place at about 36 h post-invasion, yielding schizont stages progressively containing up to 32 nuclei [10]. Merozoites are individualized around each nucleus in the very last hours of the erythrocytic cycle, just prior to their release from infected erythrocytes [10]. Protein synthesis, trafficking and organelle assembly to form mature merozoites take place mainly during the final 10–12 h of asexual development [11]. For these reasons, the FcB1-schizont-EST library was constructed by subtracting transcripts from highly synchronized late stages (42–48 h post-invasion) by transcripts from mixed remaining stages (0–40 h post-invasion) by suppression subtractive hybridization [7].

A pilot study of this library, limited to 50 clones, led to the identification of 40 genes over-expressed in schizont stages, including the well-known late-schizont/merozoite specific genes coding for EBA-175, CLAG/RhopH1, coronin, MSP1, MSP3, MSP6, myosin A, SERA and SERP proteins, which was in good agreement with the proposed stage specificity of this library [7]. Seven inserts randomly selected from this FcB1-schizont-EST library were used to probe cDNAs amplified from rings, early trophozoites, late trophozoites, early schizonts and late schizonts and all seven genes were consistently detected in samples corresponding to early and late schizonts as compared to a control gene expressed in all stages [7]. Molecular analysis of the PfDYN2 gene identified during this pilot study [PF10_0360] also confirmed its expression in late schizonts [12]. In addition to *P. falciparum *genes over-expressed in late schizont stages, this pilot study led to the identification of two genes (PF11_0494 and PFL0290w) whose EST sequences indicated intron/exon boundaries that differed from those previously predicted and four genes (CLAG/RhopH1/PFC0120w, MSP3/PF10_0345, PF13_0053 and PF14_0175) for which clear strain-dependent polymorphism was identified between FcB1 and 3D7 [7]. In this context, it was thus of interest to conduct a large-scale analysis of this FcB1-EST library. The expectations were: 1) to obtain an exhaustive collection of genes selectively over-expressed in late schizont stages; 2) to validate or invalidate *P. falciparum *gene models by aligning all ESTs with the genome sequence, and 3) to identify genes indicating protein polymorphism between the FcB1 and 3D7 strains. While writing this article, version 5.4 of PlasmoDB was released, taking into account three new sets of *P. falciparum *ESTs, namely those analyzed by Watanabe *et al*. [13] and Lu *et al*. [14] as well as the ESTs sequenced in the present work. This article presents and discusses the FcB1-schizont-EST data and its contribution to our knowledge of *P. falciparum *gene structure and polymorphism.

## Results and discussion

### FcB1-schizont-ESTs clustering on the *P. falciparum *genome

**Figure 1:**
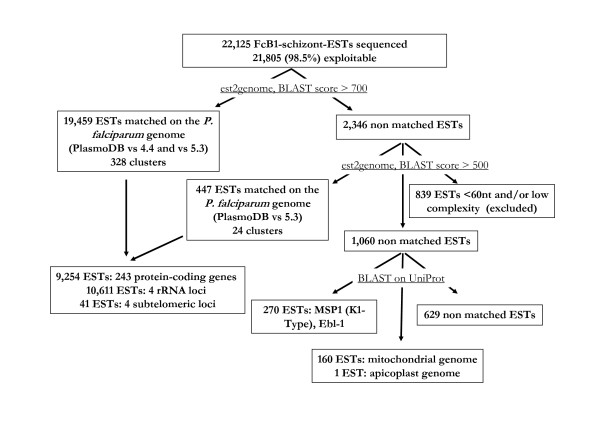
**Clustering strategy for the analysis of FcB1-schizont-ESTs**. The first two clusterings performed using est2genome (BLAST score > 700) on the 3D7 genome (PlasmoDB versions 4.4 and 5.3) allowed clustering of 19,459 ESTs. By lowering the BLAST score to 500, 447 additional ESTs were clustered and mapped on the 3D7 genome (PlasmoDB vs 5.3). The remaining unmatched FcB1-schizont-ESTs were analysed by comparison with the UniProt database, revealing 270 additional ESTs matching MSP1 (K1 type) and Ebl-1, 160 ESTs matching the mitochondrial genome and 1 EST matching the apicoplast genome.

A total of 22,125 clones randomly picked from the library were sequenced, yielding 21,805 (98.5%) usable ESTs that were matched to the *P. falciparum *genome using the BLAST/est2genome method [15]. Three successive est2genome clustering analyses were performed. The first clustering (BLAST score > 700) was carried out using PlasmoDB version 4.4  as a source of genomic data for *P. falciparum*. PlasmoDB version 4.4 provides, in addition to genomic sequences and gene annotations, various gene models built using algorithms such as PHAT, GeneFinder, GlimmerM and Hexamer [6]. The second clustering (BLAST score > 700) was achieved using PlasmoDB version 5.3, released on June 2007. It yielded very similar results except that alternative gene models were not accessible in this new PlasmoDB version, which prevented an in-depth analysis of some of the data (see further). These two clustering analyses allowed the matching of 19,459 ESTs (93.5%) into 328 distinct clusters, with a cluster being a group of overlapping ESTs (sharing at least 100 bp) matching a specific genomic locus (Figure 1). The number of ESTs per cluster ranged from 1 (see for example cluster_23, matching the hypothetical RESA-like gene PFB0085c) to thousands (see for example cluster_322, matching the rRNA unit on chromosome 5) (Additional file 1). The remaining unmatched 2,346 FcB1 ESTs that could correspond to gene fragments that diverge markedly between FcB1 and 3D7 strains, to genes present in FcB1 but absent from 3D7, or to genes encoded in the mitochondrial or apicoplast genomes, were clustered again on the *P. falciparum *genome (PlasmoDB version 5.3), thus lowering the BLAST score to 500. While 839 ESTs were rejected for being too small (< 60 nucleotides) or displaying very low complexity, 447 additional FcB1-ESTs could be significantly aligned with loci on the *P. falciparum *genome (3D7), revealing 24 additional clusters (Figure 1). The remaining unmatched ESTs were then aligned with protein sequences of the UniProt database [16] using the BLAST algorithm, revealing additional ESTs matching MSP1 variants of K1-type (210 ESTs on [UniProt:P04932] and 1 EST on [UniProt:A0SJF0/EMBL:DQ489588]), to Ebl-1 ([UniProt:Q8IEB6], 59 ESTs), the mitochondrial genome (160 ESTs on [EMBL:AJ276844]) and the *tufA *gene ([UniProt:Q25820], 1 EST) on the apicoplast genome [UniProt:X95276] (Figure 1). The results of the three est2genome clustering analyses on the 3D7 genome can be viewed at  and  respectively (*authorisation is required to access these website), and are discussed hereafter. Then results regarding FcB1-schizont-ESTs that did not match 3D7 genomic sequences but did match other *P. falciparum *sequences in the UniProt database are presented and discussed.

### FcB1-schizont-EST clusters matching the *P. falciparum *3D7 genome

A systematic analysis of the 352 clusters (328 + 24) matching the 3D7 genome was performed to identify and study the corresponding loci. These were distributed along the 14 *P. falciparum *chromosomes (Table 1) and corresponded to 243 coding genes (9254 ESTs, 326 clusters) and 8 non-protein-coding loci, namely: 4 distinct rRNA loci (10611 ESTs, 22 clusters) and 4 subtelomeric regions (41 ESTs, 4 clusters) (Additional file 1).

**Table 1:** Summary of the FcB1-schizont-EST distribution on the 14 *P. falciparum *chromosomes.

Chromosome (a)	Matched ESTs (b)	ESTs in protein coding genes (c)	ESTs in ribosomal loci (d)	ESTs in telomeric loci (e)	Protein coding genes identified (f)	Functionaly annotated protein coding genes (g)	Putative protein coding gene (h)	Hypothetical protein coding gene (i)	Confirmation of gene model (j)	Modification of gene model (k)	Evidence for protein polymorphism (l)
1	991	365	626	0	5	3	0	2	1	1	1
2	937	937	0	0	15	4	6	5	4	1	0
3	430	430	0	0	13	2	5	6	2	0	0
4	137	137	0	0	8	2	3	3	1	0	1
5	10,174	292	9,846	36	15	1	5	9	2	2	2
6	885	885	0	0	17	5	6	6	1	0	2
7	9,953	536	9,417	0	14	3	4	7	3	0	2
8	337	332	2	3	11	2	6	3	2	1	1
9	905	905	0	0	16	4	5	7	2	2	0
10	636	634	0	2	21	8	4	9	3	2	5
11	653	653	0	0	17	4	2	11	1	1	0
12	1,011	1,011	0	0	25	9	3	13	4	1	2
13	994	958	0	36	32	8	6	18	2	2	3
14	1,179	1,179	0	0	34	5	7	22	1	1	2

Total	29,222	9,254	19,891	77	243	60	62	121	29	14	21
Unique	19,906	9,254	10,611	41	243	30	62	121	29	14	21
%		46.5	53.3	0.2		25	25	50	12	6	9

This table lists the number of FcB1-schizont-ESTs (b) mapped on each of the 14 *P*. *falciparum *chromosomes (a), detailing those matching protein coding genes (c), ribosomal loci (d) or telomeric loci (e). The number of protein coding genes covered by the FcB1-schizont-ESTs is indicated in (f), (g, h, i) detailing whether these genes are functionnaly annotated genes (g), putative genes (h) or hypothetical genes (i). Columns (j, k, l) indicate the number of gene models that were confirmed (j), modified (k) or identified as displaying some strain dependent polymorphism between FcB1 and 3D7 (l) after analysis of 3D7-genomic versus FcB1-EST alignments. "Total" corresponds to the total number of cases for the 14 chromosomes, "Unique" accounts for the fact that many ribosomal-RNA-matching ESTs matched both chromosomes 5 and 7 and that the 36 ESTs matching the telomeric end of chromosome 5 also matched the telomeric end of chromosome 13.

### Protein coding genes

Among the 243 protein coding genes identified in this study, 60 (~25%) currently correspond to functionally annotated genes, 62 (~25%) to putative genes and 121 (~50%) to hypothetical genes (Table 1). Functionally annotated genes in PlasmoDB are genes whose annotations are supported by experimental data (molecular studies, biochemical characterizations, etc.) while putative genes are annotated based on significant similarities with functionally annotated genes in other species but lack experimental data in *P. falciparum *to support these annotations. Finally, hypothetical genes lack similarities with functionally annotated genes in the database and some of them rely solely on mathematical algorithms for identification. As expected, this new collection of 243 protein coding genes includes all 40 protein coding genes initially identified from the FcB1-schizont-EST library after the pilot study [7] (Additional file 1).

Available transcriptomic profiles [17-19] were recovered for each of these 243 protein coding genes and plotted on graphs to check whether the stage specificity of the FcB1-schizont-library was in agreement with the results of these other transcriptomic studies (Additional file 2). Good concordance was observed, though the FcB1-schizont collection appeared to be slightly younger than expected, corresponding mostly to genes peaking at 37–42 h in the Bozdech transcriptomic study and in early schizonts in the Le Roch transcriptomic study. This shift may be due to differences in the time required to accomplish a full erythrocytic cycle *in vitro *(42 to 48 h), which is known to be strain dependent [19]. It may also be due to slight variations in morphological appreciation of the different developmental stages. Nonetheless, most genes were confirmed by both transcriptomic studies, with few exceptions (Additional file 1). The expression of about 80% of genes of the FcB1-schizont collection reported to peak in gametocytes according to the Le Roch transcriptomic studies, appeared to peak at 37–46 h in the Bozdech transcriptomic studies (Additional file 1). Finally, conflicting expression profiles may correspond to genes differentially regulated in FcB1 as compared to 3D7, as previously observed by Llinas *et al*. for a few genes of HB3 and Dd2 strains [19]. Therefore, the FcB1-schizont-EST collection appears to be of interest as a complementary dataset for studying merozoite morphogenesis.

The FcB1-schizont-EST collection contained well known merozoite specific proteins such as merozoite capping protein-1 (PF10_0268), merozoite surface proteins MSP1 (PFI1475w), MSP3 (PF10_0345), MSP4 (PFB0310c), MSP5 (PFB0305c), MSP6 (PF10_0346), MSP7 (PF13_0197), MSP7-like (PF13_0193) and MSP9/ABRA (PFL1365c), GLURP (PF10_0344), Pfemp3 (MAL13p1.405), EBA (MAL7P1.176) and EBA-181 (PFA0125c). Interestingly, we also identified four histone genes: PFF0860c (histone H2a), PFC0920w (histone H2a variant, putative), PFF0865w (histone H3), PFF0510w (histone H3, putative) reported to be involved in nucleosome structure (GO:0000786) and assembly (GO:0006334) as well as chromatin structure (GO:0000785) and assembly (GO:0031497). In addition to these four histone genes, 3 genes are also annotated in the Gene Ontology database by the term chromosome (GO:0005694): PFE0450w (chromosome condensation protein, putative), PF14_0316 (DNA topoisomerase II, putative) and PFL1930w (hypothetical protein conserved). As previously observed [7], we also identified several cytoskeleton associated proteins: myosin A (PF13_0233), myosin D (PFL1435c), coronin (PFL2460w), dynamin-1 (PF11_0465), dynamin-2 (PF10_0368) and formin-2 (PFL0925w). Several rhoptry proteins were also present in this FcB1-schizont dataset: 3 of the 5 known CLAG/RhopH1 (PFC0110w, PFC0120w, MAL7P1.229), RhopH2 (PFI1445w), RhopH3 (PFI0265c), RAP1 (PF14_0102), RAP3 (PFE0075c), RAMA (MAL7P1.208) and also PF14_0495, which is an ortholog of the TgRON2 protein secreted from the rhoptry neck portion in *Toxoplasma gondii *[20]. We also characterized the expression of MAL8P1.73, reported to be an ortholog of Ts4705, a *T. gondii *protein detected in rhoptry extracts [20], which is also known to bind AMA-1, like TgRON2 and TgRON4 [21].

To further characterize the cellular components, molecular functions and biological processes in which the genes of the FcB1-schizont dataset may be involved, GO-terms were downloaded from GeneDB (genedb.org). GO annotations were, however, available only for 159 of the 243 genes (see Additional file 1). We then used GOStat software [22] to identify GO terms over-represented in the annotations of these 159 genes as compared to the complete *P. falciparum *genome, using a p-value threshold of 0.01 (see Table 2). Over-represented cellular components were actin cytoskeleton (GO:0015629), chromosome (GO:0005694), myosin complex (GO:0016459), nucleosome (GO:0000786) and rhoptry (GO:0020008). Similarly, over-represented molecular functions included actin-binding (GO:0003779), calcium ion binding (GO:0005509) and phospholipid binding (GO:0005543). Over-represented biological processes corresponded to cell division (GO:0051301), cytokinesis (GO:0000910), DNA packaging (GO:0006323), nucleosome assembly (GO:0006334) and entry into host cells (GO:0030260). These GO terms are in good agreement with our current knowledge of biological and molecular mechanisms that occur during merozoite morphogenesis. Conversely, a single GO term appeared to be under-represented in our study: the term defense response (GO:0006952) (see Table 2). In *P. falciparum*, this term has been attributed to *var *genes (see amigo.geneontology.org), which encode PfEMP1 surface proteins responsible for antigenic variation [23]. Since these *var *genes are known to be transcribed early during parasite development [23,24], the corresponding ESTs were not expected to be present in the FcB1-schizont library.

**Table 2:** GO term analysis of genes spanned by FcB1-schizont-ESTs.

**GO term**	**GO ID**	**Genes in the FcB1-schizont collection**	**Genes in the whole genome**	**p-value**
**Cellular Components**				

nucleosome	GO:0000786	4	8	0.000334
actin cytoskeleton	GO:0015629	5	18	0.00136
myosin complex	GO:0016459	3	6	0.00208
chromosome	GO:0005694	7	45	0.00561
rhoptry	GO:0020008	2	3	0.00694

**Biological Processes**				

entry into host cell	GO:0030260	4	7	0.000174
nucleosome assembly	GO:0006334	4	12	0.00202
cytokinesis	GO:0000910	2	2	0.00239
cytoskeleton organisation and biogenesis	GO:0007010	8	56	0.00533
DNA packaging process	GO:0006323	5	25	0.00637
cell division	GO:0051301	2	3	0.00694
cell motility	GO:0006928	2	3	0.00694
defense response	GO:0006952	1	187	0.00745

**Molecular Functions**				

actin binding	GO:0003779	6	14	2.74e-05
phospholipid binding	GO:0005543	3	7	0.0035
calcium ion binding	GO:0005509	10	86	0.00853

A statistical analysis was performed to identify GO terms over- or under-represented in the annotated genes of this collection as compared to their distribution in the annotated genes of the whole genome. GOStat software [22] was used for this analysis, using a p-value threshold of 0.01. Only 159 genes have GO annotations among the 243 genes of the collection (3241 genes have GO annotations throughout the whole genome). This table reports all non-redundant over- or under-represented terms (i.e. all over- or under-represented GO terms that do not generalize another over- or under-represented term) in the three ontologies (cellular component, biological process, and molecular function). All terms were over-represented except for the term "defense response" (biological process) that was under-represented.

Such a statistical analysis of GO-terms is still, however, limited by the number of *P. falciparum *proteins not yet annotated in the Gene Ontology database. For example, only two of the 10 rhoptry proteins that were identified in the FcB1-schizont list (see above) were annotated by the corresponding GO term (GO:0020008): RAP1 (PF14_0102) and RAP3 (PFE0075c).

### Examination of FcB1-3D7 alignments

Although most FcB1-schizont ESTs do not span the full length of the corresponding genes, a detailed systematic comparative analysis between FcB1 EST sequences and corresponding 3D7 genomic sequences was performed to check the accuracy of the currently proposed gene models in PlasmoDB and also to identify protein polymorphism between these two strains.

### Validation and modification of intron/exon boundaries

In most cases (~75%), FcB1-schizont-ESTs matched protein coding genes in the middle of described exons, without providing any relevant information for gene model validation. On several occasions (40 genes in total), FcB1-schizont-ESTs matched 5' or 3' borders of genes or spanned exon-exon boundaries. A gene by gene analysis of these cases allowed confirmation of one or several introns in 29 gene models (26 in Table 3, 3 in Table 4) and to propose modifications in 14 gene models (Table 4). Clustering of FcB1-schizont-ESTs in PlasmoDB (version 4.4), which displays alternative gene models, was particularly informative during this analysis and a few remarkable examples of gene model validations and gene model modifications are illustrated in Additional files 3 and 4, respectively. The most spectacular gene model correction was found for PFE0240w, whose FcB1-schizont-EST data provided evidence of four additional exons and an extended C-terminal end (Additional file 4C).

**Table 3:** Gene models confirmed by FcB1-schizont-ESTs.

[Gene] (a)	[Product Description] (b)	[Pf-iRBC max expr time (GS array)] (c)	[Pf-iRBC+Spz+ Gam max expr stage (Affy)] (d)	# of ESTs (e)	confirmed in gene model (f)
PFA0110w	ring-infected erythrocyte surface antigen	46	Merozoite	229	intron 1

MAL13P1.103	hypothetical protein, conserved	34	Gametocyte	1	introns 1 to 4 and exons 1 to 5 *

MAL7P1.108	hypothetical protein, conserved		Early Schizogony	7	intron 3

MAL7P1.153	hypothetical protein, conserved	46	Gametocyte	2	intron 1

MAL7P1.229	Cytoadherence linked asexual protein	40		133	intron 1

PF08_0075	60S ribosomal protein L13, putative	14	Early Trophozoite	29	intron 1

PF10_0211	hypothetical protein	42	Late Schizogony	44	intron 2

PF10_0268	merozoite capping protein 1	41	Early Schizogony	8	intron 1

PF10_0372	Antigen UB05	37	Early Schizogony	7	introns 1 and 3

PF11_0348	hypothetical protein	37	Gametocyte	29	intron 1

PF13_0173	hypothetical protein, conserved	42	Late Schizogony	58	intron 1

PF14_0429	RNA helicase, putative	43	Early Ring	16	exon 1 and intron 1

PFB0310c	merozoite surface protein 4		Early Schizogony	33	intron 1

PFB0340c	cysteine protease, putative	37	Early Schizogony	582	introns 2 and 3

PFB0475c	hypothetical protein, conserved	46	Late Schizogony	40	introns 1 and 2

PFB0815w	Pf Calcium-dependent protein kinase 1	42	Late Schizogony	60	introns 1 to 4 *

PFC0120w	Cytoadherence linked asexual protein, 3.2	40	Early Schizogony	252	introns 1 to 5 *

PFC0920w	histone H2A variant, putative	39	Late Schizogony	42	introns 1 and 2

PFD0940w	hypothetical protein, conserved	38	Early Schizogony	52	intron 2

PFE1415w	cell cycle regulator with zn-finger domain, putative	40	Gametocyte	10	introns 5 to 8 *

PFF0185c	hypothetical protein	41		21	intron 14

PFI0265c	RhopH3	41	Early Schizogony	183	exons 4, 5, 6

PFI1445w	High molecular weight rhoptry protein-2	39	Early Schizogony	95	introns 1 and 6

PFL0975w	hypothetical protein, conserved	38	Early Schizogony	27	introns 3 and 4 and end of gene *

PFL1160c	hypothetical protein, conserved	39	Early Schizogony	31	introns 2 and 3

PFL2505c	hypothetical protein, conserved		Late Schizogony	107	introns 1 and 2

This table, derived from Additional file 1, lists the 26 *P. falciparum *genes whose gene models were confirmed by FcB1-schizont-ESTs. The first 4 columns were downloaded from PlasmoDB version 5.3 and correspond respectively to: gene accession numbers in PlasmoDB (a), their current description in PlasmoDB (b), the maximum expression time during the erythrocytic cycle according to the transcriptomic data of [17,19] (c) and the maximum expression stage according to the transcriptomic data of [18] (d). Column (e) indicates the number of ESTs corresponding to each gene and isolated in this study. Column (f) details the genetic elements that were confirmed. Asterisks (*) in this last column refer to examples illustrated in Additional file 3. Pf-iRBC, *P. falciparum*-infected red blood cells.

**Table 4:** Gene models modified by FcB1-schizont-ESTs.

[Gene] (a)	[Product Description] (b)	[Pf-iRBC max expr time (GS array)] (c)	[Pf-iRBC+Spz+Gam max expr stage (Affy)] (d)	# of ESTs (e)	modified in gene model (f)
PFA0630c	hypothetical protein	16	Early Trophozoite	17	in agreement with chr1.genefinder_16r, chr1.glimmerm_366 and chr1.phat_146 *

MAL13P1.460	conserved hypothetical protein			77	intron 3 modified

MAL8P1.73	hypothetical protein, conserved	40	Early Schizogony	28	intron 16 modified but intron 18 confirmed

PF10_0072	hypothetical protein		Late Schizogony	8	exon 1 would be longer at 3' end

PF10_0361	hypothetical protein	23	Early Ring	37	in agreement with chr11, glimmer_1141

PF11_0194	hypothetical protein	41	Gametocyte	50	in agreement with chr11, genefinder.157r *

PF13_0193	MSP7-like protein		Early Schizogony	41	exon 1 would be longer at 3' end

PF14_0280	phosphotyrosyl phosphatase activator, putative	20	Gametocyte	2	gene would be longer downstream

PFB0305c	merozoite surface protein 5	46	Late Schizogony	5	exon 2 would be longer at 5' end

PFE0240w	hypothetical protein, conserved		Gametocyte	12	four additional exons, longer protein *

PFE1490c	hypothetical protein, conserved		Early Ring	16	intron 1 modified but intron 2 confirmed

PFI0905w	hypothetical protein		Gametocyte	4	exon 2 would be longer at 5' end **

PFI1565w	conserved protein		Late Schizogony	11	3'-end of gene in agreement with chr9.glimmerm_973 and chr9.glimmerm_974 *

PFL0290w	hypothetical protein, conserved	43	Early Trophozoite	11	intron 1 modified but intron 2 confirmed *

This table, derived from Additional file 1, lists the 14 *P. falciparum *genes whose gene models were corrected based on FcB1-schizont-ESTs. Columns (a) to (e) are as described in the Table 3 legend. The last column (f) details each modification. Note that three genes in this list were both modified/confirmed (in different parts): MAL8P1.73, PFE1490c and PFL0290w. Asterisks (*) in this last column refer to examples illustrated in Additional file 4. The model revision proposed for PFI0905w (**) will need to be confirmed by other experimental data since these 4 ESTs also matched *P. falciparum *telomerase RNA (see Additional file 10). Pf-iRBC, *P. falciparum*-infected red blood cells.

### FcB1 versus 3D7 protein polymorphism for 21 protein coding genes

Although the FcB1-schizont-EST collection provides useful nucleotide sequence data to indicate single nucleotide polymorphism (SNP) specific to the FcB1 strain, these data (which will be available in PlasmoDB) are not discussed here. However, during the systematic comparative analysis of FcB1-schizont-ESTs versus 3D7 sequences, we identified 21 genes for which some protein polymorphism was observed between FcB1 and 3D7 encoded proteins (Table 5). As illustrated in Additional file 5 where these 21 protein alignments are displayed, in most cases, this protein polymorphism corresponded to a variable number of repeated elements or to amino acid variations in these repeat elements. Malaria protein polymorphism has been suggested to be one of the main strategies of the parasite to evade the host immune mechanism, and antigens that are under natural immune pressure tend to have a higher level of polymorphism [25,26]. These 21 genes therefore seem to be good candidates for encoding such antigens. However, according to PlasmoDB, only 4 of these 21 genes encode proteins harbouring putative signal peptides consistent with their exposure on cell surface: PF10_0177 (erythrocyte membrane-associated antigen), PF10_0345 (MSP3), PF10_0351 (hypothetical protein expressed in late schizogony) and PFL1385c (MSP9/ABRA). For the other proteins, these results raise questions about the reasons underlying such polymorphism or how these proteins may be exported either to the surface of the parasite or the surface of infected-red blood cells [27].

**Table 5:** Evidence of protein polymorphism between FcB1 and 3D7 strains.

[Gene] (a)	[Product Description] (b)	[Pf-iRBC max expr time (GS array)] (c)	[Pf-iRBC+Spz+Gam max expr stage (Affy)] (d)	# of ESTs (e)	type of polymorphism (f)
PFA0215w	hypothetical protein, conserved	45	Late Schizogony	72	in tandem repeats

PFD0185c	peptidase	42	Gametocyte	10	in tandem repeats

PFE0250w	hypothetical protein, conserved	25	Early Trophozoite	39	in Asn-rich region

PFE0655w	hypothetical protein, conserved	16	Early Trophozoite	2	in tandem repeats

PFF0670w	hypothetical protein, conserved	38		47	in Asn-rich region

PFF0765c	hypothetical protein, conserved	41		3	in tandem repeats

MAL7P1.208	rhoptry-associated membrane antigen, RAMA	41		147	in tandem repeats

PF07_0111	hypothetical protein, conserved	37	Gametocyte	1	in tandem repeats

PF08_0109	hypothetical protein, conserved	39	Early Schizogony	5	in Asn-rich region

PF10_0177	erythrocyte membrane-associated antigen	40	Gametocyte	31	in tandem repeats

PF10_0184	hypothetical protein	41	Gametocyte	13	local polymorphism

PF10_0213	10b antigen, putative	33	Early Schizogony	20	in Asn-rich region

PF10_0345	merozoite surface protein 3	42	Late Schizogony	93	mild polymorphism

PF10_0351	hypothetical protein	45	Late Schizogony	83	in tandem repeats

PFL0465c	Zinc finger transcription factor (krox1)		Late Schizogony	1	mild polymorphism

PFL1385c	Merozoite Surface Protein 9, MSP-9	41	Early Schizogony	310	mild polymorphism

PF13_0053	hypothetical protein, conserved	12	Early Trophozoite	52	in tandem repeats

MAL13P1.158	hypothetical protein, conserved	40	Gametocyte	10	local polymorphism

PF13_0245	hypothetical protein, conserved	46	Early Trophozoite	16	mild polymorphism

PF14_0175	conserved protein unknown function			107	in tandem repeats

PF14_0486	elongation factor 2	17	Early Trophozoite	5	mild polymorphism

This table, derived from Additional file 1, lists the 21 *P. falciparum *genes for which some protein polymorphism was identified between FcB1 and 3D7 strains. Columns (a) to (e) are as described in the Table 3 legend. The last column (f) details the various cases. All protein sequence alignments are illustrated in Additional file 5. Pf-iRBC, *P. falciparum*-infected red blood cells.

### 3D7 non-protein coding loci covered by FcB1-schizont-ESTs

Due to the methodology used to build the FcB1-schizont-EST library, i.e. isolation of total RNA followed by selective conversion of polyA+ RNA into double strand cDNA by SMART-PCR [7], a very large number of sequences (10,611) corresponded to rRNA fragments, as previously observed [7]. These non-protein coding, rRNA-matching ESTs represent more that half of the sequenced ESTs, which is much higher than the 30%, estimated after the pilot study [7]. This may be due to the fact that all picked clones were randomly sequenced in this current high throughput study, while in the pilot study EST clones had been size selected prior to sequencing.

The rRNA gene organisation in the *P. falciparum *genome is very unusual as compared to other eukaryotes. Instead of having hundreds of repetitions of rRNA units in its genome, *P. falciparum *has seven complete and incomplete rRNA (18s-5.8s-28s) units on six of its 14 chromosomes and 3 copies of 5s rRNA genes on chromosome 14 [5] (Table 6). Some of these rRNA loci are known to be expressed in a developmentally regulated fashion, e.g. the two A-type rRNA units (on chromosomes 5 and 7) that are mainly expressed in human infection stages and the two S-type rRNAs (on chromosomes 11 and 13) that are expressed in insect infection stages [5,28]. The role played by other rRNA loci present in the *P. falciparum *genome is not entirely clear [5].

**Table 6:** Summary of the FcB1-schizont EST distribution on the different rRNA loci of *P. falciparum*.

rRNA type	Chromosomal location ^(a)^	rRNA gene structure	Expression if known	Number of Matching ESTs	Corresponding clusters
A-type	Chromosome 5 (1,290 kb–1,296 kb)	18s – 5.8s – 28s	Human	9846 ^(f)^	322, 328, 303, 302, 323, 324, 325, 326, 327

A-type	Chromosome 7 (1,139 kb–1,146 kb)	18s – 5.8s – 28s ^(b)^	Human	9417 ^(f)^	54, 35, 37, 40, 44, 46, 49, 52, 55

S-type	Chromosome 11 (1,926 kb–1,933 kb)	18s – 5.8s – 28s	Insect	None	

S-type	Chromosome 13 (2,796 kb–2,800 kb)	18s – 5.8s – 28s	Insect	None	

Not defined	Chromosome 1 (457 kb–482 kb)	18s – 5.8s – 28s ^(c)^	Unknown	626	146, 147, 148

Not defined	Chromosome 8 (93 kb–100 kb)	28s – tmp2 ^(d)^	Unknown	None	

Not defined	Chromosome 8 (1,281 kb–1,289 kb)	5.8s – tmp1 ^(d)^	Unknown	2	62

Not defined	Chromosome 14 (779 kb–781 kb)	5s ^(e)^	Unknown	None	

(a) Approximate position indicated in kb; (b) also contains atypical 18s and 28s; (c) 18s and 5.8s are of S-type and 28s is divergent (65% A-type and 75% S-type); (d) 18s is missing from these units; (e) 3 tandem repeats of 5s. Data compiled from [5] and PlasmoDB. (f) Most of these ESTs clustered with homologous loci of both chromosomes 5 and 7.

### Most non-protein coding FcB1-schizont-ESTs correspond to the two A-type rRNA units on chromosomes 5 and 7

In this study, a very large majority of the rRNA-matching ESTs clustered on both A-type rRNA units, located on chromosomes 5 and 7 (Table 6) and most matched both homologous A-type regions on chromosomes 5 and 7 (Additional file 6). However, a limited number of FcB1-schizont ESTs clustered on two atypical rRNA units, respectively located on chromosome 1 (626 ESTs) and chromosome 8 (2 ESTs) (Table 6). None of the FcB1-schizont EST clusters matched any of the two S-type rRNA loci, which was an interesting negative control of our data regarding the developmental stage of the studied parasite population.

### FcB1-schizont-EST analysis provides evidence of the actual expression of two atypical rRNA loci located on chromosomes 1 and 8

A total of 626 ESTs matched chromosome 1 at the level of MAL1_28s, which is annotated as A-Type rRNA in PlasmoDB but described as being atypical (65% identity to A-type and 75% identity to S-type) by Gardner *et al*. [5]. The four longest ESTs matching this locus did not match elsewhere in the *P. falciparum *genome (Additional file 7). These results indicated that the MAL1_28s gene was indeed expressed in our experimental population of highly synchronized FcB1 parasites. Owing to the small number of truly specific ESTs corresponding to MAL1_28s, the expression level of this gene in the parasite population is obviously much lower than the level rRNA gene expression from typical Chr5 and Chr7 A-type loci (Table 6). While the physiological conditions allowing MAL1_28s rRNA expression remain to be determined, our experimental data clearly argue in favour of the actual expression of MAL1_28s RNA which, to the best of our knowledge, has not yet been documented.

The two ESTs matching chromosome 8 were mapped on a non-annotated area located between MAL8a_5.8s and PF08_temp1 (Additional file 8), with the latter also being annotated as rRNA encoding in PlasmoDB. Both are non-protein coding ESTs and do not match elsewhere in the genome. This strongly suggests that they are indeed encoded by this new locus of unknown function in which, interestingly, several SNPs are indicated (see PlasmoDB version 5.4). The FcB1-schizont-EST data therefore supports the transcription of a non-protein coding RNA at this new locus of chromosome 8.

### Non-protein coding FcB1-schizont-ESTs matching sub-telomeric regions

Four clusters (cluster_304, Chr05_01; cluster_64, Chr08_01; cluster_98, Chr10_01 and cluster_188, Chr13_01) and one atypical EST (PU0AAA27YL11RM1, Chr07_15) matched sub-telomeric regions in the *P. falciparum *genome, very close to the chromosome ends (see Additional file 1). Notably, the 36 ESTs corresponding to cluster_304 (Chr05_01) also corresponded to cluster_188 (Chr13_01). An in-depth analysis of these ESTs, using the BLAST algorithm optimized for highly similar sequences (via NCBI BLAST server), indicated that they matched virtually all chromosome ends (90 to 93% identity at the nucleotide level, data not shown), in telomere-associated repeat element (TARE) regions [29,30]. To refine this analysis, these ESTs were aligned to the nucleotide sequence corresponding to a particularly well annotated telomeric end on chromosome 3 [EMBL:AL034560]. These FcB1-schizont-ESTs were then found to match the same region, likely corresponding to the TARE1 or TARE2 region, between telomere and R-CG7 segments [31] (Additional file 9).

### Non-protein coding FcB1-schizont ESTs matching telomerase RNA

An independent analysis of the FcB1-schizont-EST library aimed at unravelling the genetic structure of the *P. falciparum *telomerase RNA (TR-RNA) revealed 5 FcB1 ESTs that mapped to the 5' end of the recently described telomerase RNA gene in a template binding region [32] (Additional file 10). It was then noted that four of these FcB1-schizont-ESTs also corresponded to the cluster 500_89 cluster, matching the hypothetical gene PFI0905w (see Additional file 1). The fact that these were non-protein coding ESTs indicates that they corresponded to the telomerase RNA gene rather than to cDNA fragments of the PFI0905w gene.

### In-depth analysis of FcB1-schizont-ESTs not matching the 3D7 genome reveals similarities to other variants of the MSP1 gene and to a paralog of EBA-140 (MAL13P1.60) and EBA-175 (MAL7P1.176)

The MSP1 gene codes for one of the most polymorphic proteins in *P. falciparum*. The analysis of FcB1-schizont-ESTs using est2genome allowed clustering of 139 ESTs with the N-terminal end of the MSP1 gene in PlasmoDB (PFI1475w, cluster_78 and cluster_79). However, additional FcB1-schizont-ESTs matching other MSP1 variants were discovered by comparing unmatched ESTs to the UniProt database using the BLAST algorithm (Figure 1). 210 of these FcB1-schizont-ESTs matched five partly overlapping regions of the K1-type MSP1 [UniProt:P04932] and 1 additional ESTs matched the C-terminal end of MSP1 in an Iranian isolate [EMBL:DQ489588] reported to be of K1-type [33]. Therefore, a total of 350 FcB1-schizont-ESTs spanned about 50% of the FcB1-MSP1 gene (see Additional file 11). Interestingly, in the pilot study of this library, we found that the MSP3 variant in FcB1 was also of K1-type [7].

Comparison of FcB1-schizont-ESTs with the UniProt database also revealed 59 ESTs matching [UniProt:Q8IEB6]. These ESTs partly spanned the C-terminal end of the protein (amino acids 1646 to 2188) with 93 to 100% identity. [UniProt:Q8IEB6] is annotated as Ebl-1 from the strain 3D7 but, in fact, two *P. falciparum *proteins appear as paralogs of [UniProt:Q8IEB6]: EBA-175 (MAL7P1.176, BLAST e-value = 6.7 e-83) and EBA-140 (MAL13P1.60, BLAST e-value = 5.7e-82). We thus believe that these ESTs correspond to a FcB1 gene belonging to the EBL family, known to be involved in invasion [34].

### A few FcB1-schizont ESTs mapped on organellar genomes

FcB1-schizont-ESTs matching the mitochondrial genome [UniProt:AJ276844] were mapped at the level of the three described genes: putative cytochrome oxidase III [UniProt:Q9MDY3], putative cytochrome oxidase I [UniProt:Q02766] and putative cytochrome b [UniProt:Q02768]. The single EST matching the apicoplast genome [UniProt:X95276], corresponded to the *tufA *gene [UniProt:Q25820]. This latter result, based on a single EST in the whole library corresponding to transcription of the apicoplast genome, which is known to occur just prior to the transcription of merozoite specific genes [17], provides an interesting control of the proposed stage specificity of this FcB1-EST library.

## Conclusion

Extensive analysis of the FcB1-schizont-EST library, which was constructed to be enriched in transcripts over-expressed in the last hours of the parasite development in erythrocytes, provided valuable information regarding genes expressed in the late stages of the parasite development. Concerning the stage specificity, both the pilot study including experimental controls [7] and the current work provide support that the genes spanned by these ESTs were actually expressed late during the parasite development, even though in many cases this conclusion was based on comparative analysis with other transcriptomic studies [17-19]. The fact that a single EST (out of a total of 21,805) matched the apicoplast genome, further indicates that all genes corresponding to this FcB1-schizont are expressed later, at least in the FcB1 strain. The transcriptomic profiles determined by [17] and [18] for the 243 genes of this FcB1-schizont collection indicate, however, that these genes may be expressed slightly earlier than initially expected: 37–42 h (early schizogony) rather than 42–48 h (late schizogony). This observation is in line with the fact that we identified 10 genes coding for rhoptry proteins and only two coding for micronemal proteins (EBA (MAL7P1.176) and EBA-181 (PFA0125c)). Indeed, it has been shown that rhoptry proteins are expressed prior to micronemal proteins during merozoite morphogenesis [11]. For all of these reasons, we believe that this FcB1-schizont collection is a truly appropriate dataset to focus on early steps of merozoite morphogenesis after apicoplast transcription. The 121 proteins currently annotated as hypothetical or hypothetical conserved are therefore favourable candidates involved in this morphogenesis. One shortcoming is, however, the fact that in several cases no additional experimental data is currently available to confirm the expression of these candidates. Additional data and molecular analyses are thus required to confirm the involvement of these candidates in merozoite morphogenesis as, for example, was done in the case of PfDYN2 [12]. But, interestingly, the total number of genes identified in the FcB1-schizont dataset (243) is consistent with the number of genes proposed by [17] as corresponding to merozoite specific genes.

An interesting outcome of this study is that intron/exon boundaries were validated in 29 genes, revised gene models were proposed for 14 genes and FcB1 versus 3D7 protein polymorphisms were identified for 21 genes. Although this information was mostly collected at the gene fragment rather than full-length gene level, it represents a valuable contribution, along with similar data by others [13,14], to gain greater insight into *P. falciparum *gene structure and polymorphism.

The most unexpected results concerned ESTs corresponding to non-protein coding RNAs present in this FcB1-schizont collection. Although these ESTs are limited in number, especially as compared to the very high number of ESTs matching the well known A-type rRNA loci, they provided indications on the actual expression of a 28s rRNA gene located on chromosome 1 and on the expression of a non-protein coding transcript between MAL8a_5.8s and Pf08_tmp1 genes. ESTs providing this information do not cluster elsewhere in the *P. falciparum *genome, so they are strictly specific to these two mainly uncharacterized loci. The actual physiological conditions under which these loci are expressed are, however, totally unknown. A second small series of 41 non-protein coding ESTs provided information regarding the actual transcription of TARE regions, likely corresponding to TARE-1 or TARE-2. To the best of our knowledge, this has not been shown before. Finally, 5 ESTs matched the 5' end of *P. falciparum *telomerase RNA. Rather than indicating that this RNA is expressed, which has recently been reported [32], this result raises the question as to the structure and expression of PFI0905w, located at the very same genomic locus.

The methodology used to built the FcB1-schizont-EST library, i.e. suppressive subtractive hybridization [35], which was selected to ensure stage specificity, has some technical disadvantages. The first is the very high redundancy in the ESTs produced, which is directly due to the PCR-amplification step. The second is the fact that these ESTs are necessarily digested by an endonuclease to be subtracted (here using *Rsa*I) and are therefore of small or very small size. Consequently, most genes identified by this strategy were only partly spanned by FcB1-schizont-ESTs. These shortcomings did not affect the analyses in the pilot study [7] and were therefore not expected to limit the present work. Nevertheless, the high throughput analysis of the FcB1-schizont-EST library affected the full-length coverage of gene loci by EST clusters and was biased by EST multicopies. For example, in the case of MSP1 and based on the results of the pilot study, we were expecting to yield enough ESTs to span the entire gene, but, instead, we obtained a large number of highly redundant ESTs at fragmented locations. Despite this low full-length gene coverage, the in-depth analysis of this FcB1-schizont-EST collection should help, together with data by others [13,14], to gain further insight into *P. falciparum *gene structure and polymorphism. In the present work, which involved a high-throughput analysis of this FcB1-schizont-EST library, a few unexpected gene expression features were also discovered, such as the actual transcription of atypical rRNA loci and subtelomeric regions.

## Methods

### Library construction

The library construction, by suppressive subtractive hybridization, was previously published [7]. Briefly, the chloroquine-resistant *P. falciparum *FcB1/Colombia strain was cultured and synchronised using standard methods. Two specific populations were isolated: a highly synchronized late stage population corresponding to late schizont/merozoite stages (42–48 h post-invasion, with schizonts containing at least 4 nuclei) and a reference population containing a homogeneous distribution of rings, trophozoites and early schizonts (containing at most 3 nuclei). Parasites were isolated from these two populations using a 0.2% saponin/1 × PBS treatment and total RNA was purified, treated by RQ1 RNase-free DNase (Promega) and converted to high quality cDNAs using the SMART (switch mechanism at the 5'-end of reverse transcript) PCR cDNA synthesis methodology, as recommended (Clontech). Subtracted cDNA populations were then generated using the PCR-Select procedure [35] according to the manufacturer's instructions (Clontech), with slight modifications [7]. Digestion with *Rsa*I yielded cDNA fragments with an average size of ~580 bp. These subtracted cDNAs fragments (i.e. ESTs), corresponding to transcripts over-expressed in late schizont/merozoite stages, were subsequently ligated into pT-Adv (Clontech) and transformed into *Escherichia coli *TOP10F' competent cells (Clontech).

A total of 22,125 randomly picked clones were sequenced by the rolling circle sequencing procedure, using universal primers, at Genoscope. Raw sequences were treated to mask the various primers used to construct the subtracted library (SMART primer, NP1 and NP2 primers, Clontech), small sequences were removed yielding 21,805 reads, 87.2% of which read through the complete insert. Correspondence between EMBL/GenBank/DDBJ accession numbers, PU accession numbers and clusters described in this current work are provided in Additional file 12.

### Alignment of FcB1-schizont EST sequences on the 3D7 *P. falciparum *genome

The *P. falciparum *genomic data was imported from PlasmoDB . In this current work, we used PlasmoDB versions 4.4 and 5.3. We used a two-step strategy to align EST sequences on the *P. falciparum *genome. As a first step, BLAST [36] was used to align microsatellite repeat-masked EST sequences and genomic sequences using the following settings: W = 20, X = 8, match score = 5, mismatch score = -4. The sum the HSP (high-scoring pair) scores was then calculated for each possible location, and the location with the highest score was then retained if the sum of scores was more than 700. Once the location of the transcript sequence was determined, the corresponding genomic region was extended by 5 kb on each side. Transcript sequences were then realigned on the extended region using EST_GENOME [15] (mismatch 2, gap penalty 3) to define transcript exons [37]. These transcript models were fused by a single linkage clustering approach whereby transcripts from the same genomic region sharing at least 100 bp are merged [38].

FcB1-schizont-EST sequences have been released to the EMBL/GenBank/DDBJ under the accession numbers [EMBL:CU657981] to [EMBL:CU672219].

The clustering of FcB1-schizont-ESTs on the *P. falciparum *genome (PlasmoDB version 4.4) can be viewed at:  (authorisation required) [39]. The clustering of the FcB1-schizont-ESTs on the *P. falciparum *genome (PlasmoDB version 5.3) can be viewed at: . (authorisation required)

## Abbreviations

AMA-1: apical membrane antigen; bp: base pair(s); BLAST: basic local alignment sequence tool; cDNA: complementary DNA(s); CDS: coding sequence(s); CLAG: cytadherence linked antigen; EBA: erythrocyte binding antigen; EBL: erythrocyte binding like; EST(s): expressed sequence tag(s); GLURP: glutamate rich protein; GO: Gene Ontology; MSP: merozoite surface antigen; ORF: open reading frame(s); PCR: polymerase chains reaction; PfEMP1/3: *P. falciparum *erythrocyte membrane protein 1/3; PHAT: pretty handy annotation tool; RAMA: Rhoptry associated membrane antigen; RAP: rhoptry-associated protein; RESA: ring-infected erythrocyte surface antigen; Rhop: Rhoptry; RON: rhoptry neck; rRNA: ribosomal RNA; SERA: serine repeat antigen; SERP: serine repeat protein; SMART-PCR: switch mechanism at the 5'-end of reverse transcript-polymerase chain reaction; SNP: single nucleotide polymorphism; SSH: suppressive subtracting hybridization; TARE: telomere-associated repeat element; TR-RNA: telomerase RNA.

## Authors' contributions

IF project design, collaborator contacts, analyses of data alignment, interpretation of results, manuscript, figures and tables writing; BMP *P. falciparum *genomic and EST data recovery, treatment, est2genome clustering, data formatting and graphical interface set-up; EG alignment data analyses and interpretations, participation to text and figures writing; CDS *P. falciparum *genomic and EST data treatment, est2genome clustering, UniProt BLAST searches, data formatting for EMBL submissions and publication, contribution to figures; FA bioinformatics supervision, *P. falciparum *genomic and EST data recovery and treatment, est2genome clustering, data formatting for EMBL submissions and publication; EM alignment data analyses, interpretation of results, participation to text and figures writing; LB and OG GO-term recovery and statistical analysis, participation to text and figures writing; SC subtracted library construction; PW: sequencing supervision, *P. falciparum *genomic and EST data recovery and treatment, est2genome clustering, data formatting for EMBL submissions and publication; PG: project design, interpretation of results. All authors read and approved the manuscript.

## Supplementary Material

Additional file 1 **Complete list of the 253 genomic loci of 3D7 spanned by FcB1-schizont-ESTs**. (a, b, c and d) were downloaded from PlasmoDB (version 5.3) and correspond, respectively, to the gene accession numbers in PlasmoDB (a), their current description in PlasmoDB (b), the maximum expression time during the erythrocytic cycle according to the transcriptomic data by [17,19] (c) and the maximum expression stage according to transcriptomic data by [18] (d). (e, f) list the different loci according to their occurrence along each chromosome (from chromosome 1 to 14) and their approximate localisation on each chromosome, in kilobase pairs (f). The corresponding clusters (g) and the total number of ESTs (h) are also indicated. In column (i), we indicate whether the FcB1-EST data allowed us to confirm or modify gene models, or revealed some protein polymorphism and column (j) details these confirmations/modifications/indications. (NI) stands for "non-informative". In (k), Gene Ontology accession numbers, downloaded from GeneDG (genedb.org) are indicated. Genes previously identified in the pilot study [7] are indicated by "yes" in column (l). Color code: red is for functionally annotated genes, blue for putative genes, black for hypothetical genes, green for non-protein coding loci. Click here for file

Additional file 2 **Stage distribution of protein coding genes spanned by FcB1-schizont-ESTs according to other microarray studies**. Comparisons were performed with the transcriptomic data of Bozdech *et al*. [17] and Le Roch *et al*. [18], respectively. A: number of protein coding genes covered by FcB1-schizont-ESTs, according to their maximum expression time during *P. falciparum *development in erythrocytes, in hours [17]; B: number of protein coding genes covered by FcB1-schizont-ESTs, according to their maximum expression stage: 1 (early rings), 2 (late rings), 3 (early trophozoites), 4 (late trophozoites), 5 (early schizonts), 6 (late schizonts), 7 (merozoites), 8 (gametocytes) [18]. C and D correspond to the same data, while taking into consideration the total number of individual FcB1-schizont-ESTs per protein coding gene. Pf-iRBC, *P. falciparum*-infected red blood cells. Click here for file

Additional file 3 **Remarkable examples of gene model validations**. These examples are indicated by * in Table 3. For each example, the scale on top indicates kilobases along the chromosome (which is mentioned above the blue line), FcB1-schizont-ESTs are symbolized below (coloured boxes for exons, arrows for introns) while also indicating the cluster number and location (thin green line). All PlasmoDB gene models, as downloaded from vs 4.4, are indicated below (green boxes for exons, arrows for introns). The various confirmed gene models are: A: MAL13P1.103 (Chr13_08), hypothetical protein conserved (introns 1 to 4 and exons 1 to 5); B: PFB0815w (Chr02_15), Pf calcium-dependent protein kinase 1 (introns 1 to 4); C: PFC0120w (Chr03_02), cytoadherence linked asexual protein (introns 1 to 5); D: PFE1415w (Chr05_15), cell cycle regulator with zinc-finger domain, putative (introns 5 to 8); E, PFL0975w (Chr12_09), hypothetical protein conserved (introns 3 and 4 and end of the gene). Click here for file

Additional file 4 **Remarkable examples of gene model modifications**. These examples are indicated by * in Table 4. The representations are as indicated for Additional file 3. The various modified gene models are: A: PFA0630c, hypothetical protein, (Chr01_06), FcB1-schizont-EST data (cluster_149) is in agreement with chr1. genefinder_16r, chr1. glimmer 366 and chr1. phat_146 models; B: PF11_0194, hypothetical protein, (Chr11_08), FcB1-schizont-EST data (cluster_122) is in agreement with Chr11.genefinder_157r model; C: PFE0240w, hypothetical protein, conserved, (Chr05_05), FcB1-schizont-EST data (cluster_308) modifies gene for which no prediction was available. It indicates four additional exons and predicts a longer protein (172 aa versus 115 aa); D: PFI1565w, conserved protein, (Chr09_14), FcB1-schizont-EST data (cluster_81) is in agreement with chr9.glimmerm_973 and chr9.glimmerm_974 models for the end of the gene; E: PFL0290w, hypothetical protein, conserved, (Chr12_03), FcB1-schizont-EST data (cluster_59) suggests that intron 1 would be smaller. Intron 2, however, is confirmed. Click here for file

Additional file 5 **FcB1 versus 3D7 protein polymorphism**. All 21 cases reported in Table 5 are illustrated. In each example, "FcB1" corresponds to the protein sequences deduced from a representative FcB1-schizont-EST and "3D7" to protein sequences deduced from the homologous 3D7 gene (EMBL/GenBank/DDBJ accession numbers indicated). Most sequence alignments were obtained using classical BLAST searches (default parameters) at NCBI . For Chr05_12, Chr12_15 and Chr13_12, sequence alignments were performed using the EMBOSS package available at EBI . Click here for file

Additional file 6 **List of 26 FcB1-schizont-ESTs representative of the 9983 FcB1-ESTs matching rRNA loci on chromosomes 5 and 7**. To limit this redundancy in the public databases, 26 sequences corresponding to the longest ESTs on each cluster were selected to be deposited in EMBL. (a) PU number of the EST; (b) size in bp; (c) corresponding rRNA element (5.8s, 18s, 28s or ITS1, internal transcribed spacer 1); (d, e, f) position of Hit on chromosome 5 (d), chromosome 7 (e) and chromosome 1 (f), respectively. Click here for file

Additional file 7 **Clustering of 626 FcB1-schizont-ESTs on the MAL1_28s gene of the chromosome 1 rRNA locus**. A: view of the chromosome 1 locus (positions 473 k to 482 k) from PlasmoDB (version 4.4); B: view of the same locus on the Genoscope browser. Only the first, longest, 14 FcB1-ESTs are shown, the remaining 612 ESTs (corresponding to cluster_146 and cluster_147) are not shown here. The four largest ESTs (PU0AAA1YC08RM1, PU0AAA22YG18RM1, PU0AAA13YE12RM1, and PU0AAA44YM17RM1, indicated by *) are strictly specific to this chromosome 1 locus. Most of the smallest ESTs also clustered on homologous rRNA loci on chromosomes 5 and 7, MAL5_28s and MAL7_28s. (see Genoscope browser for details). Click here for file

Additional file 8 **FcB1-schizont-ESTs matching the atypical rRNA locus on chromosome 8, downstream of MAL8a_5.8s and upstream of PF08_tmp1**. A: view of the chromosome 8 locus (positions 1281 k to 1293 k) from PlasmoDB (version 4.4); B. view of the same locus on the Genoscope browser. Click here for file

Additional file 9 **FcB1-schizont-ESTs matching sub-telomeric regions of chromosomes**. A: schematic representation of *P. falciparum *sub-telomeric regions and consensus sequences reported for each structure, compiled from [29] and [30]. B: alignment of representative ESTs for Chr05_01 = Chr13_01 (cluster_304 and cluster_188, PU0AAA56YB23RM1), Chr10_01 (cluster_98, PU0AAA22YJ11RM1), Chr08_01 (cluster_64, PU0AAA57YH13RM2) and Chr07_15 (cluster_48, PU0AAA27YL11RM1) on the end of chromosome 3 (EMBL AL034560), shown as a typical example, These FcB1-schizont-ESTs are localized in TARE regions, between telomere and R-CG7 segments [31]. Click here for file

Additional file 10 **FcB1-schizont-ESTs matching the template boundary element (TBE) and template regions of *P. falciparum *telomerase RNA**. The sequence of *P. falciparum *telomerase RNA (Pf TR telomerase) was aligned with sequences of the five FcB1-schizont-ESTs (PU0AAA accessions numbers indicated) by using ClustalW showing extensive conservation. Template boundary element (TBE) and template regions, both located at the 5'-end of the *P. falciparum *telomerase RNA, are as defined by [32]. Click here for file

Additional file 11 **Coverage of the MSP-1 gene by FcB1-schizont-ESTs**. MSP1 protein sequences (1720 amino acids for the 3D7-type (downloaded from PlasmoDB), 1630 amino acids for the K1-type [UniProt:P04932], 1720 amino acids for the Iranian isolate [UniProt: A0SJF0/EMBL:DQ489588] were aligned and are represented schematically, red segments corresponding to gaps. The regions covered by the 139 ESTs matching 3D7-MSP1 are indicated above the alignment, the regions covered by 210 ESTs matching K1-MSP1 below. The EST matching the MSP1 variant [UniProt:A0SJF0] (Iranian isolate of K1-type) is also represented. Cluster_78 (corresponding to 21 ESTs matching amino-acid positions 230 to 403 of 3D7-MSP1) and cluster_79 (corresponding to 118 ESTs matching the N-terminal end of the protein to position 260) are indicated as arrows. The remaining 210 ESTs matching K1-MSP1 are represented as five groups with no cluster names. For each group, the number of ESTs is indicated above each arrow and the boundaries below each arrow. Click here for file

Additional file 12 **EMBL/GenBank/DDBJ AC numbers**. Correspondence between EMBL/GenBank/DDBJ AC numbers (1^st ^column), PU numbers (2^nd ^column) and corresponding clusters or UniProt accession numbers, as described in the text (3^rd ^column), with nme standing for "non matched EST". [UniProt:A0SJF0] corresponds to Iranian-MSP1, K1-type, [EMBL:AJ276844]) corresponds to the *P. falciparum *mitochondrial genome, [UniProt:P04932] corresponds to MSP1 variants of K1-type, [UniProt:Q8IEB6] corresponds to Ebl-1, [UniProt:X95276] corresponds to the *P. falciparum *apicoplast genome. Click here for file
