# Supplementary figures and images for: A Plasmodium falciparum FcB1-schizont-EST collection providing clues to schizont specific gene structure and polymorphism
Source: BMC Genomics. 2009 May 19;10:235. doi: 10.1186/1471-2164-10-235 (PMC2695484; doi:10.1186/1471-2164-10-235)

**A**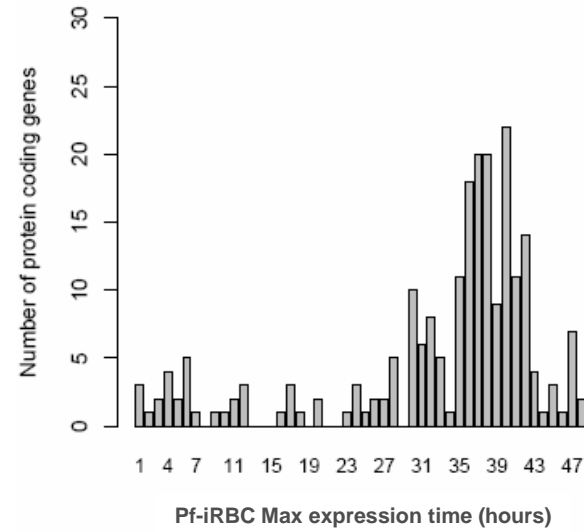**B**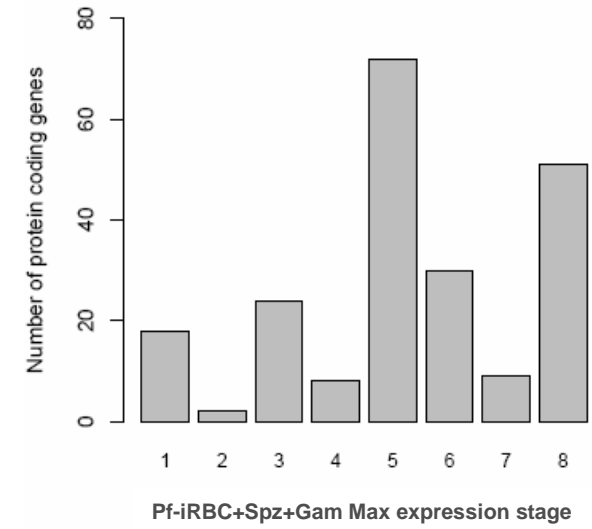**C**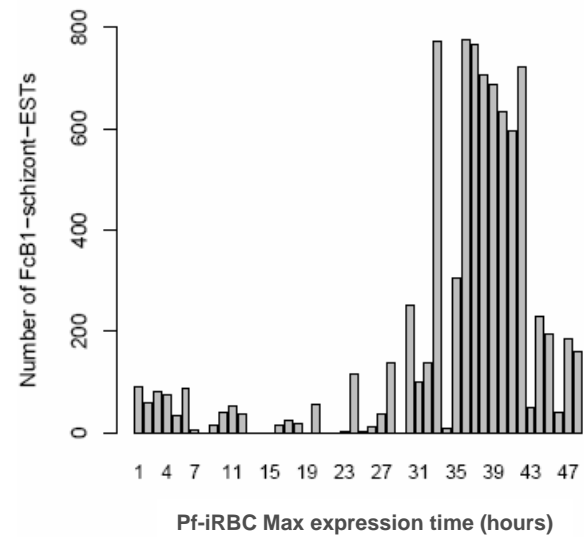**D**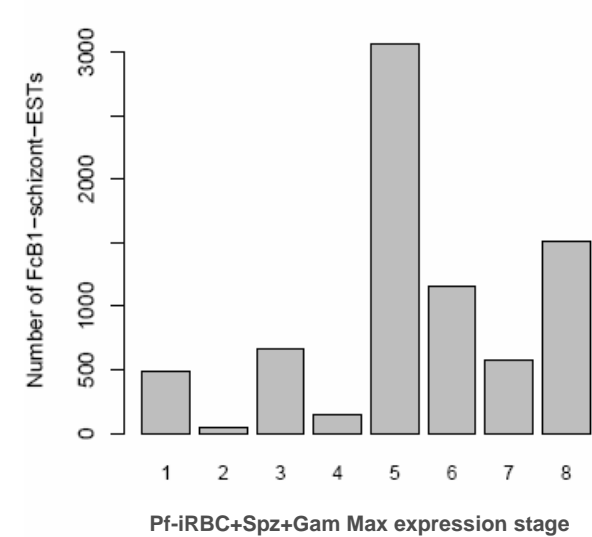

Supplement: Additional file 2 — Stage distribution of protein coding genes spanned by FcB1-schizont-ESTs according to other microarray studies. Comparisons were performed with the transcriptomic data of Bozdech et al. [17] and Le Roch et al. [18], respectively. A: number of protein coding genes covered by FcB1-schizont-ESTs, according to their maximum expression time during P. falciparum development in erythrocytes, in hours [17]; B: number of protein coding genes covered by FcB1-schizont-ESTs, according to their maximum expression stage: 1 (early rings), 2 (late rings), 3 (early trophozoites), 4 (late trophozoites), 5 (early schizonts), 6 (late schizonts), 7 (merozoites), 8 (gametocytes) [18]. C and D correspond to the same data, while taking into consideration the total number of individual FcB1-schizont-ESTs per protein coding gene. Pf-iRBC, P. falciparum-infected red blood cells. [file 1471-2164-10-235-S2.pdf]

## A, MAL13P1.103

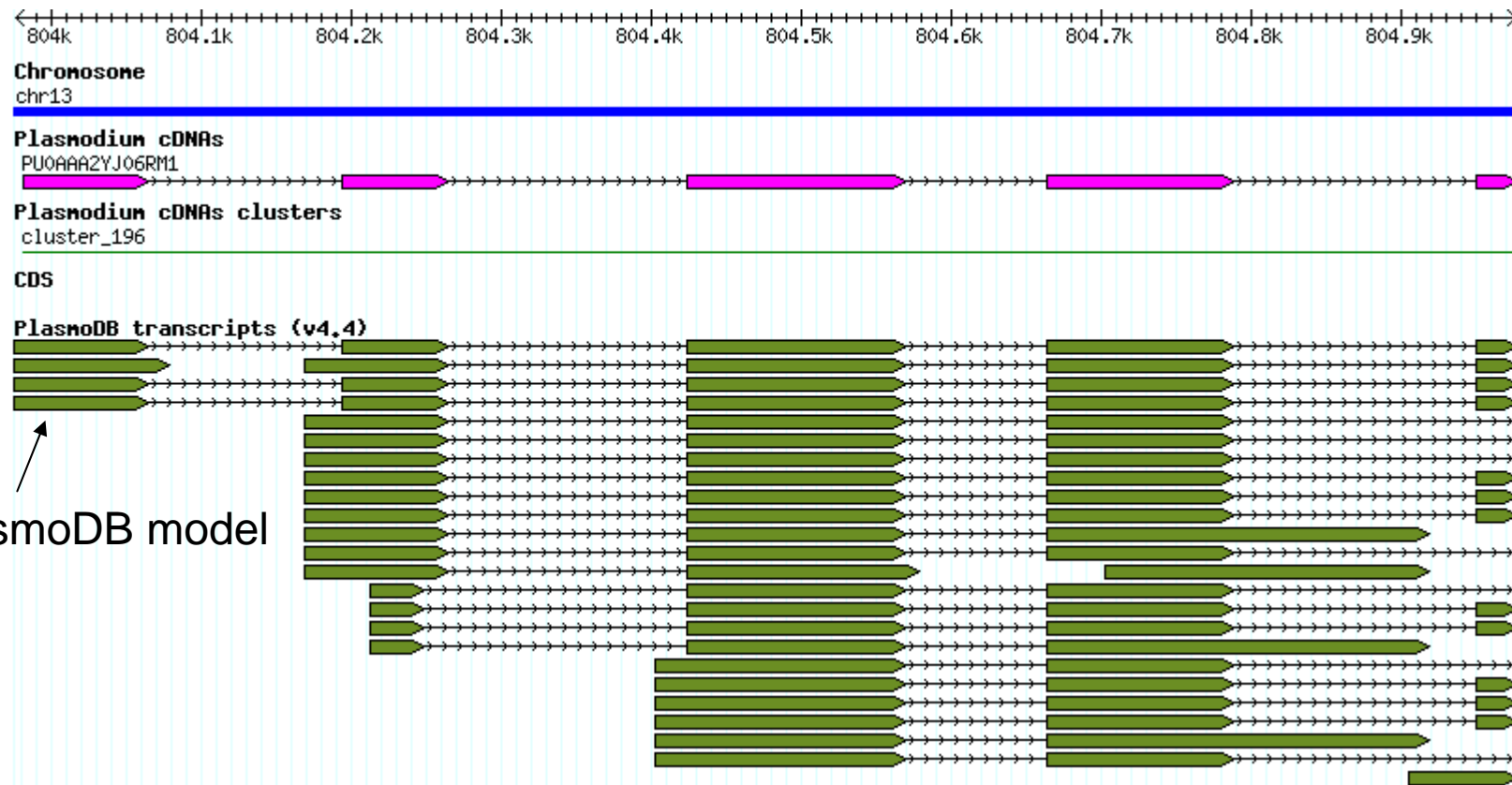

## B, PFB0815w

PlasmoDB model

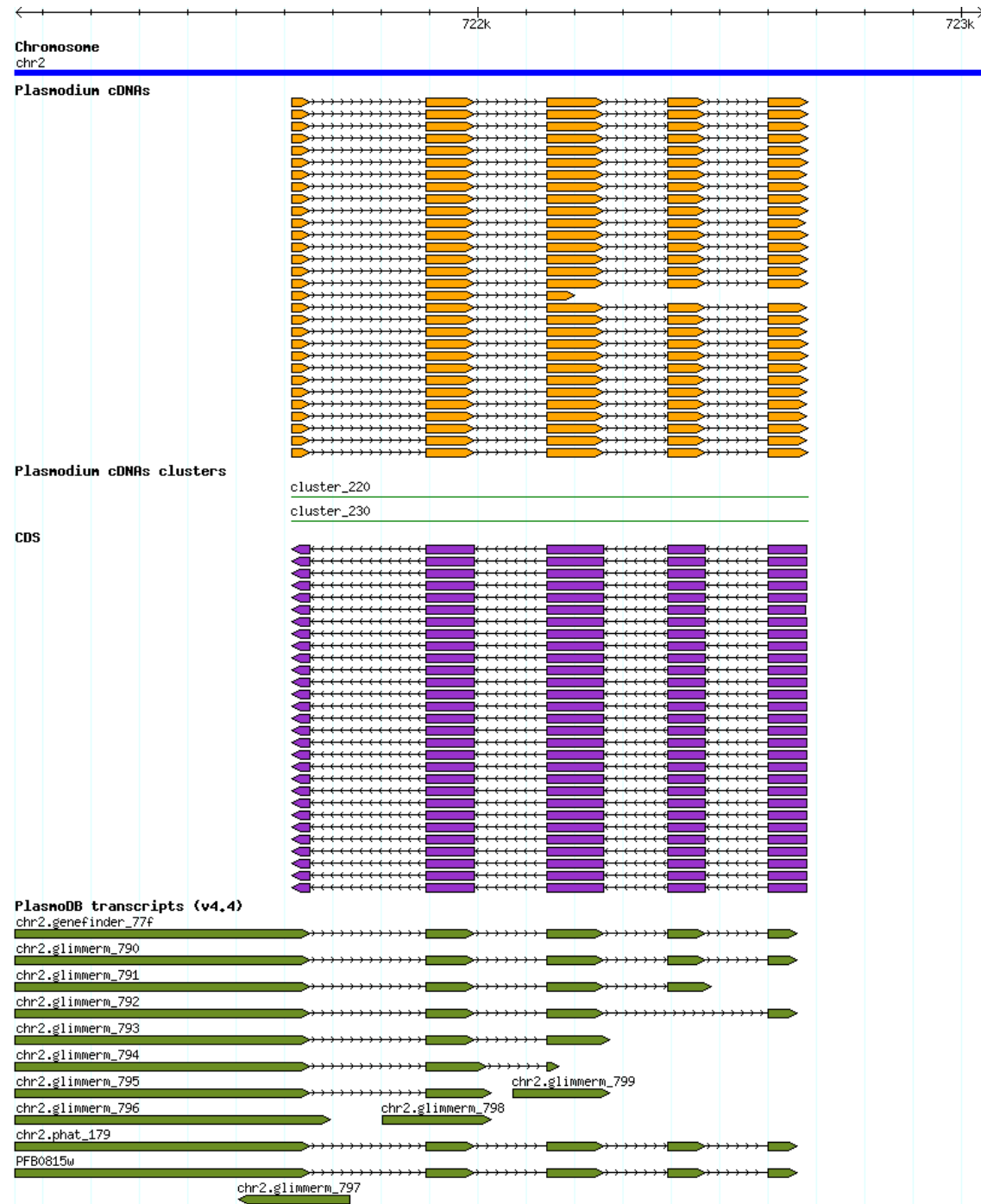

C, PFC0120w

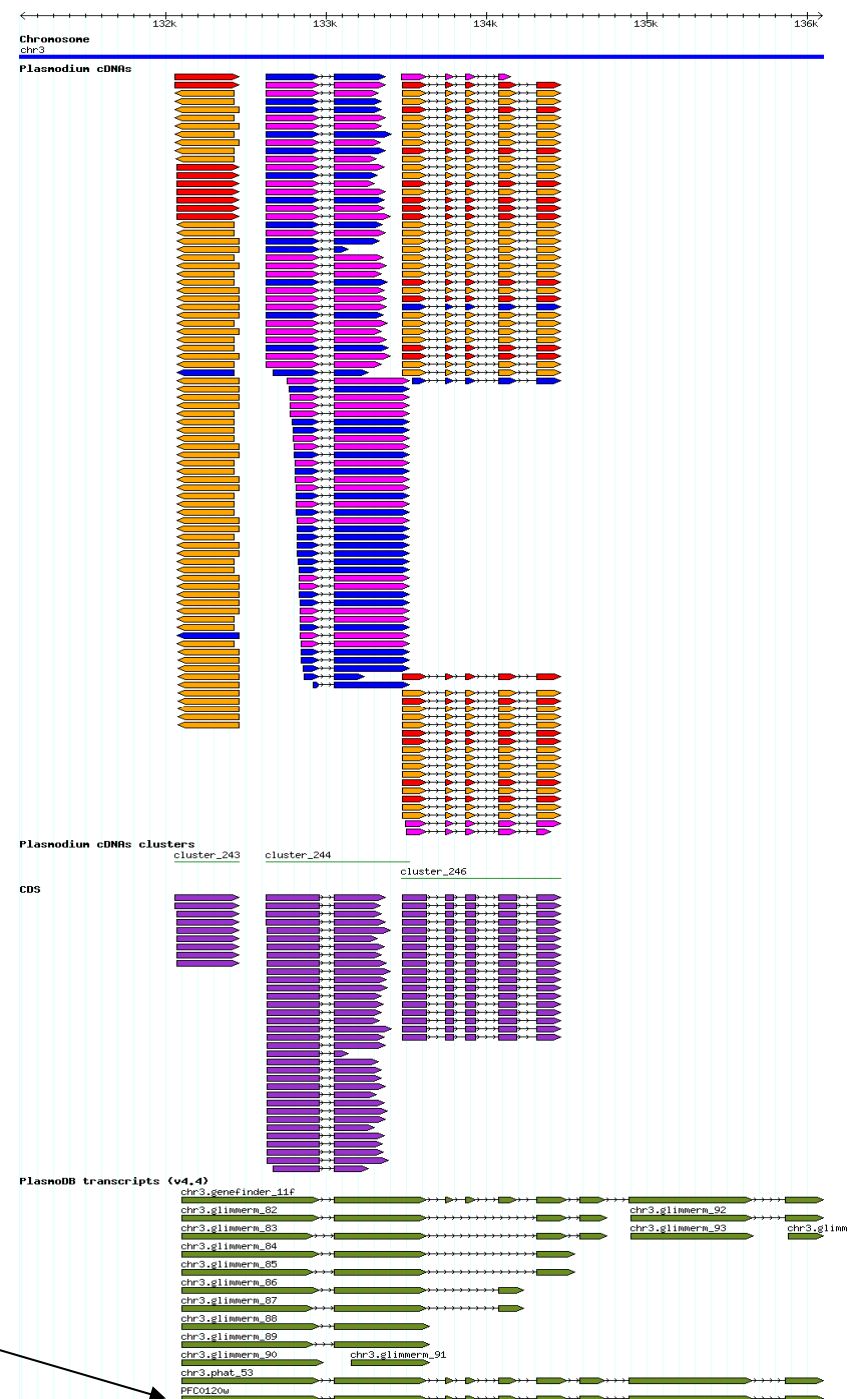

PlasmoDB model

## D, PFE1415w

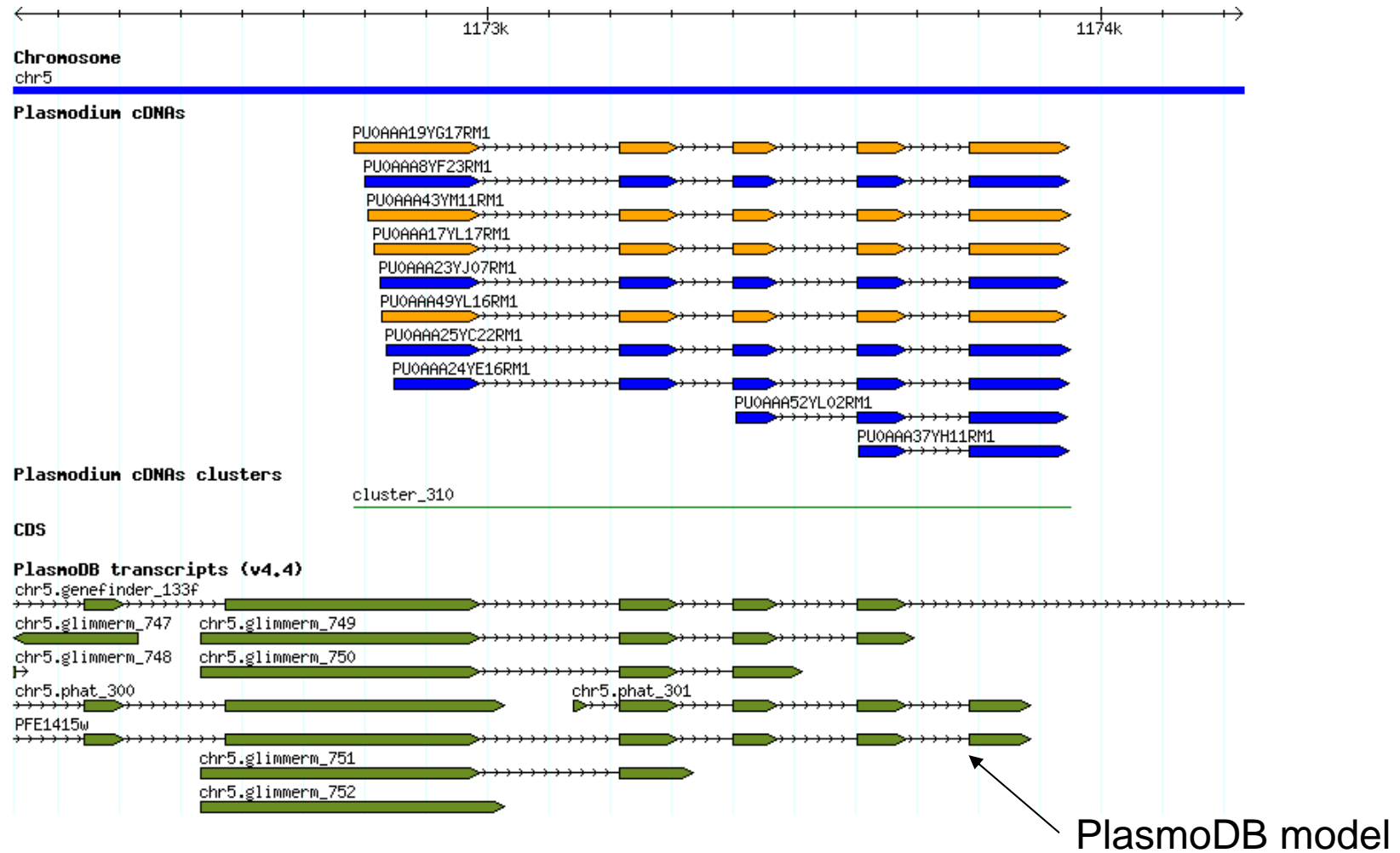

## E, PFL0975w

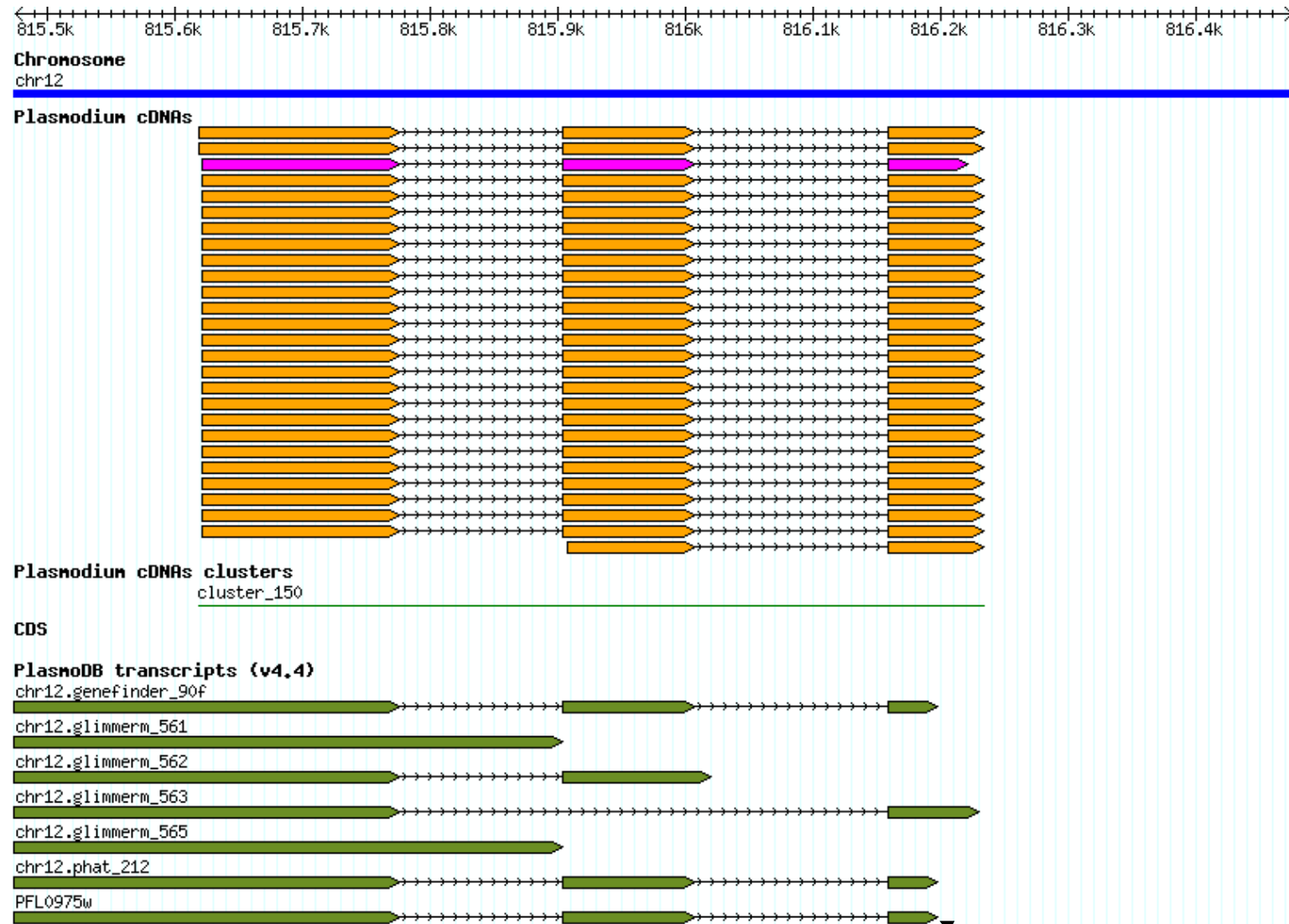

Supplement: Additional file 3 — Remarkable examples of gene model validations. These examples are indicated by * in Table 3. For each example, the scale on top indicates kilobases along the chromosome (which is mentioned above the blue line), FcB1-schizont-ESTs are symbolized below (coloured boxes for exons, arrows for introns) while also indicating the cluster number and location (thin green line). All PlasmoDB gene models, as downloaded from vs 4.4, are indicated below (green boxes for exons, arrows for introns). The various confirmed gene models are: A: MAL13P1.103 (Chr13_08), hypothetical protein conserved (introns 1 to 4 and exons 1 to 5); B: PFB0815w (Chr02_15), Pf calcium-dependent protein kinase 1 (introns 1 to 4); C: PFC0120w (Chr03_02), cytoadherence linked asexual protein (introns 1 to 5); D: PFE1415w (Chr05_15), cell cycle regulator with zinc-finger domain, putative (introns 5 to 8); E, PFL0975w (Chr12_09), hypothetical protein conserved (introns 3 and 4 and end of the gene). [file 1471-2164-10-235-S3.pdf]

A, PFA0630c

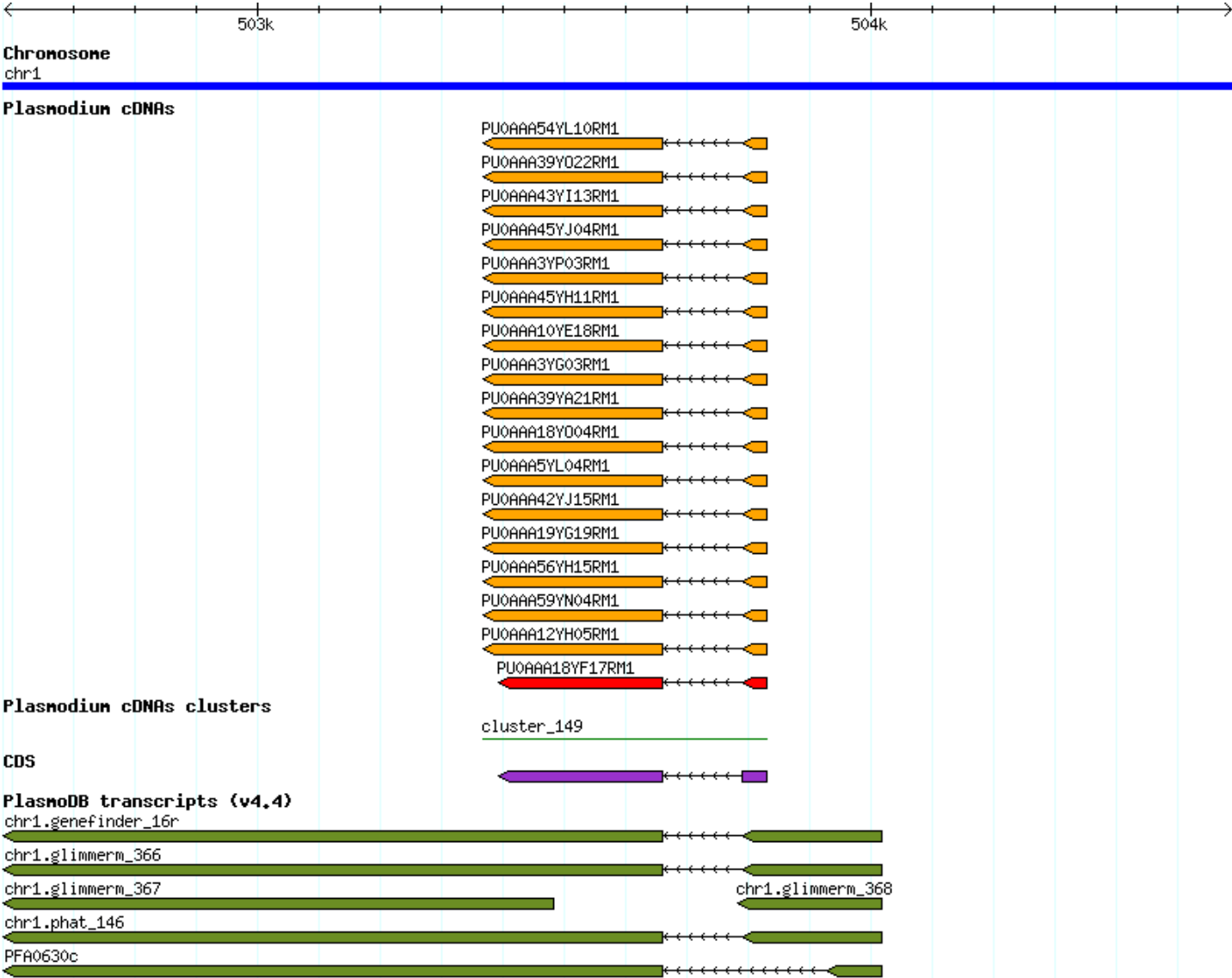

## B, PF11\_0194

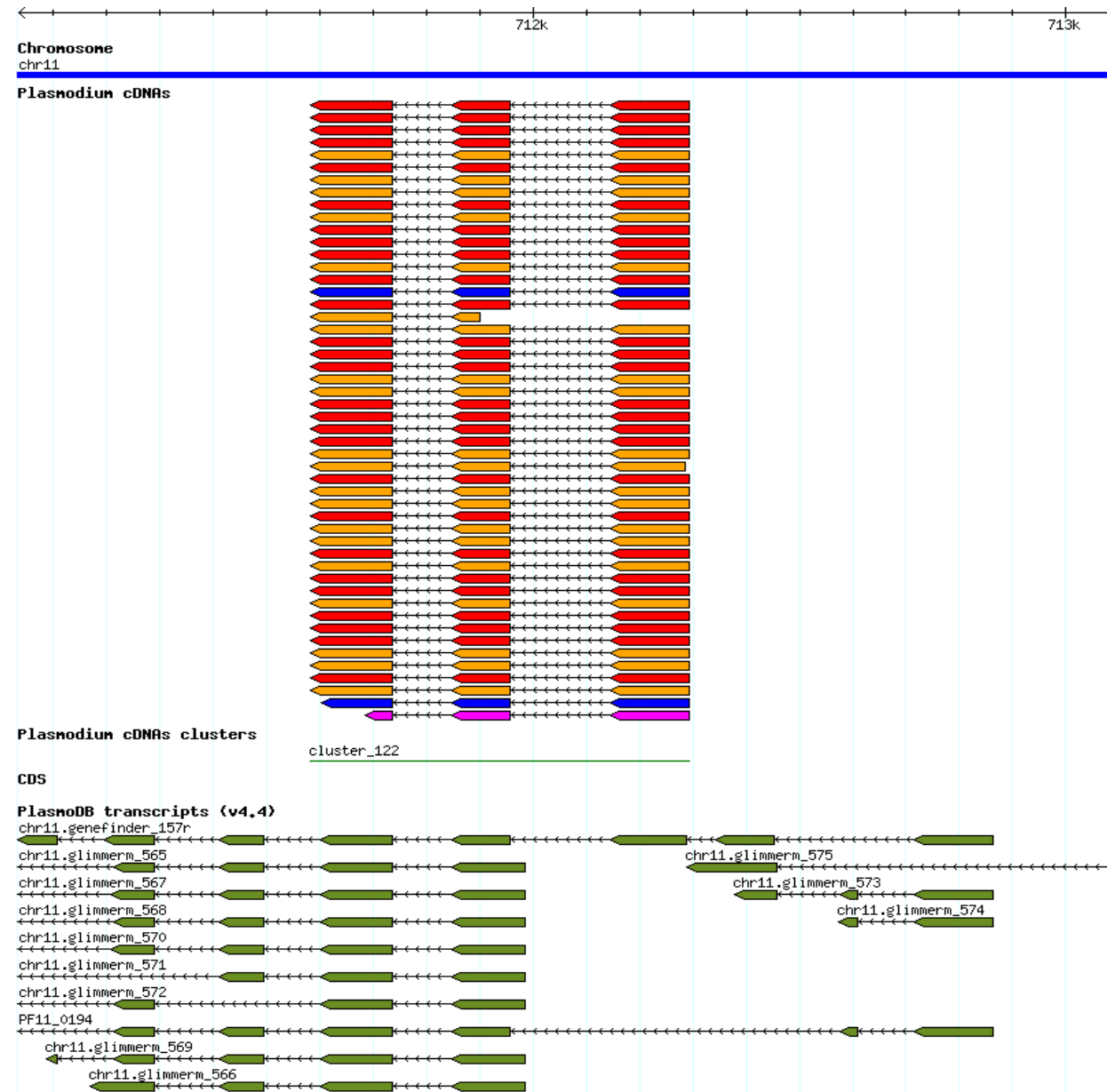

## C, PFE0240w

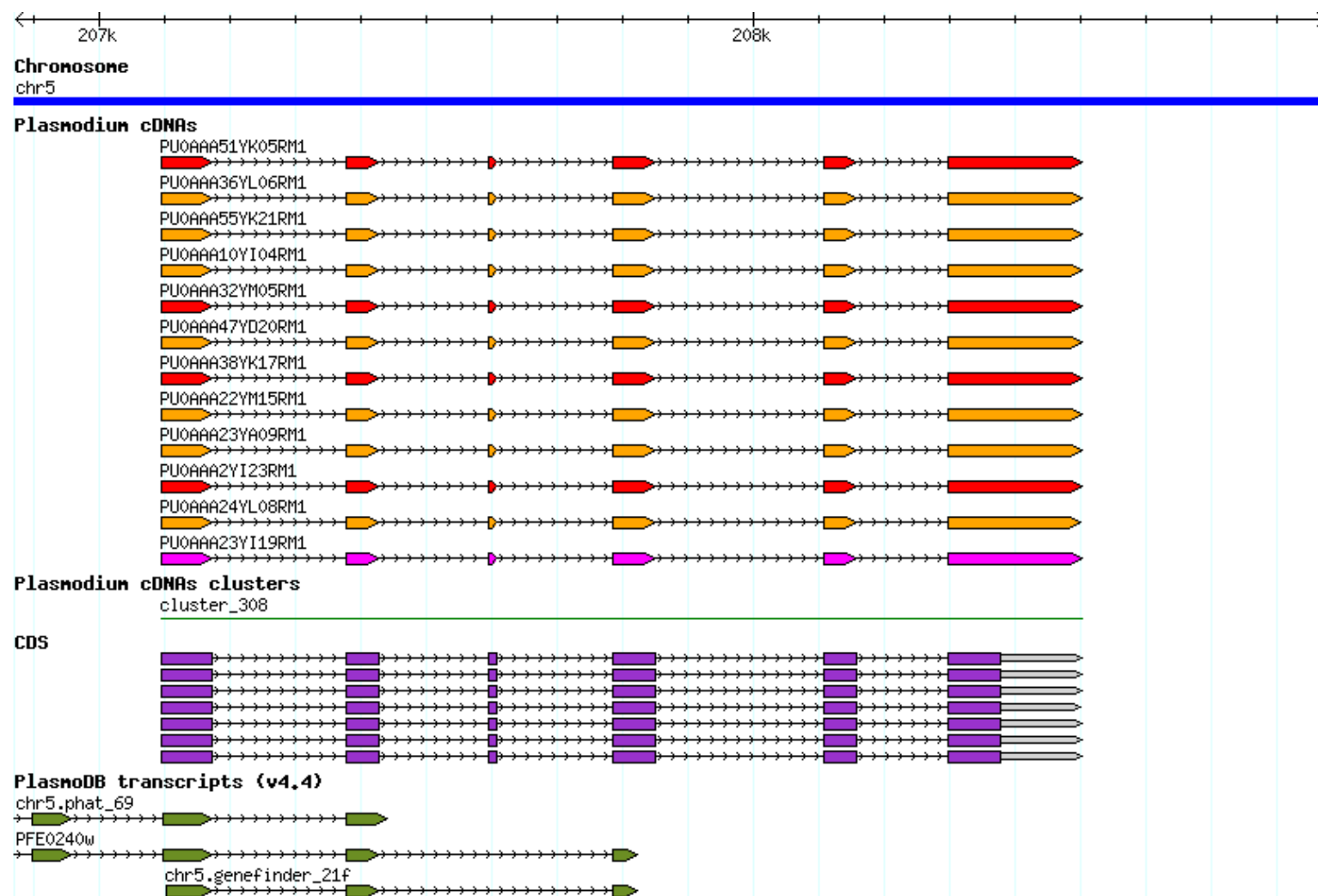

## D, PFI1565w

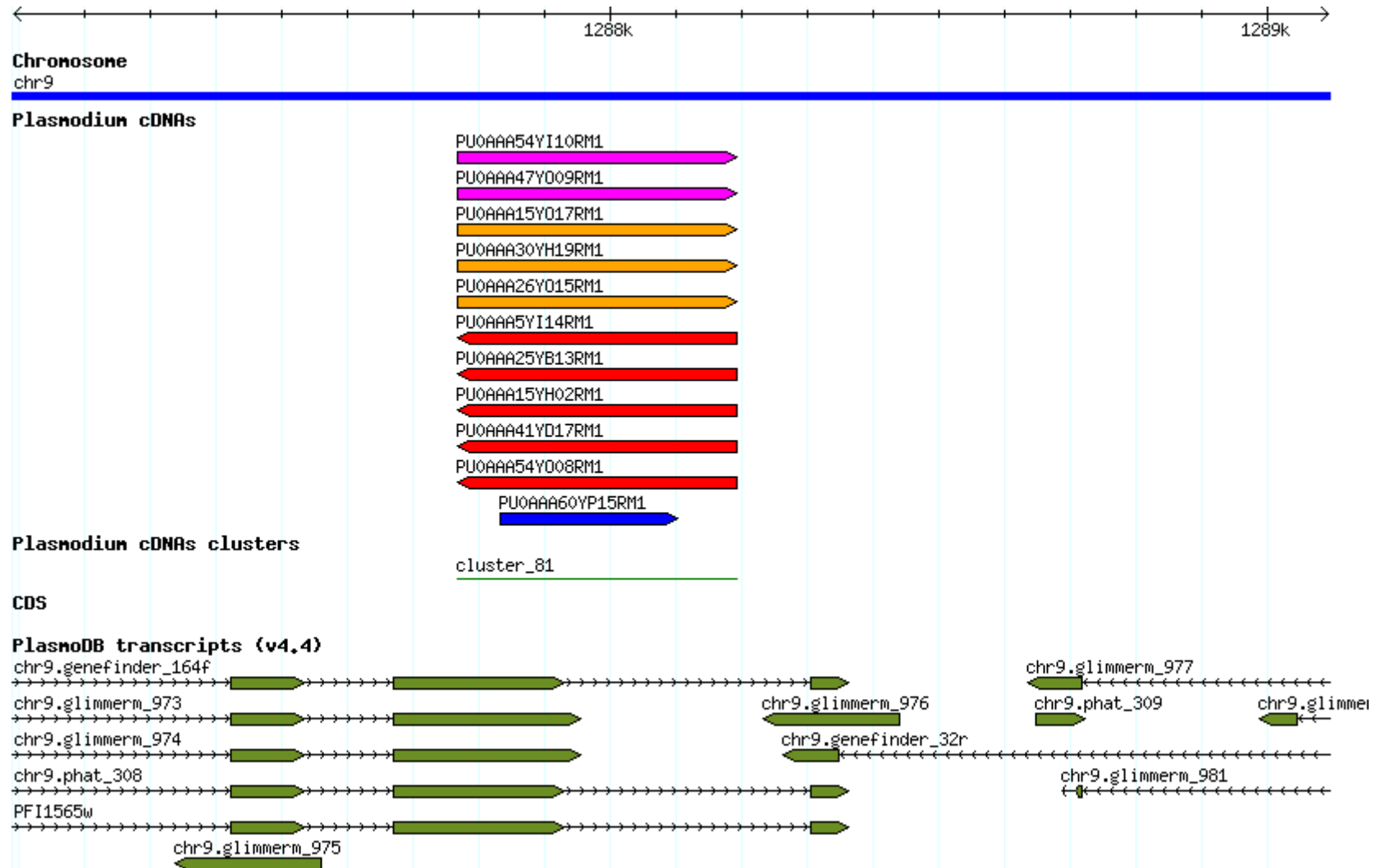

E, PFL0290w

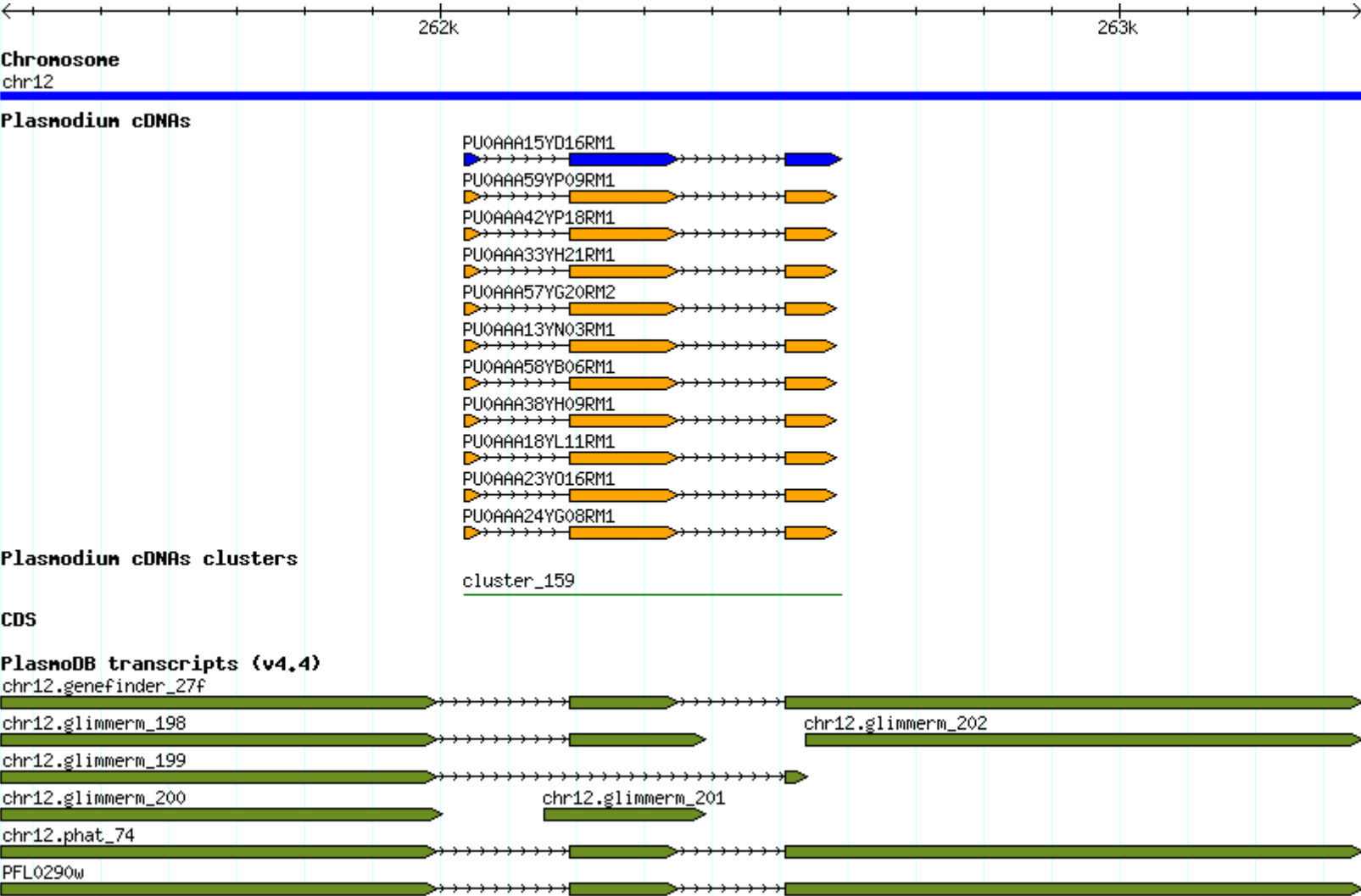

Supplement: Additional file 4 — Remarkable examples of gene model modifications. These examples are indicated by * in Table 4. The representations are as indicated for Additional file 3. The various modified gene models are: A: PFA0630c, hypothetical protein, (Chr01_06), FcB1-schizont-EST data (cluster_149) is in agreement with chr1. genefinder_16r, chr1. glimmer 366 and chr1. phat_146 models; B: PF11_0194, hypothetical protein, (Chr11_08), FcB1-schizont-EST data (cluster_122) is in agreement with Chr11.genefinder_157r model; C: PFE0240w, hypothetical protein, conserved, (Chr05_05), FcB1-schizont-EST data (cluster_308) modifies gene for which no prediction was available. It indicates four additional exons and predicts a longer protein (172 aa versus 115 aa); D: PFI1565w, conserved protein, (Chr09_14), FcB1-schizont-EST data (cluster_81) is in agreement with chr9.glimmerm_973 and chr9.glimmerm_974 models for the end of the gene; E: PFL0290w, hypothetical protein, conserved, (Chr12_03), FcB1-schizont-EST data (cluster_59) suggests that intron 1 would be smaller. Intron 2, however, is confirmed. [file 1471-2164-10-235-S4.pdf]

A

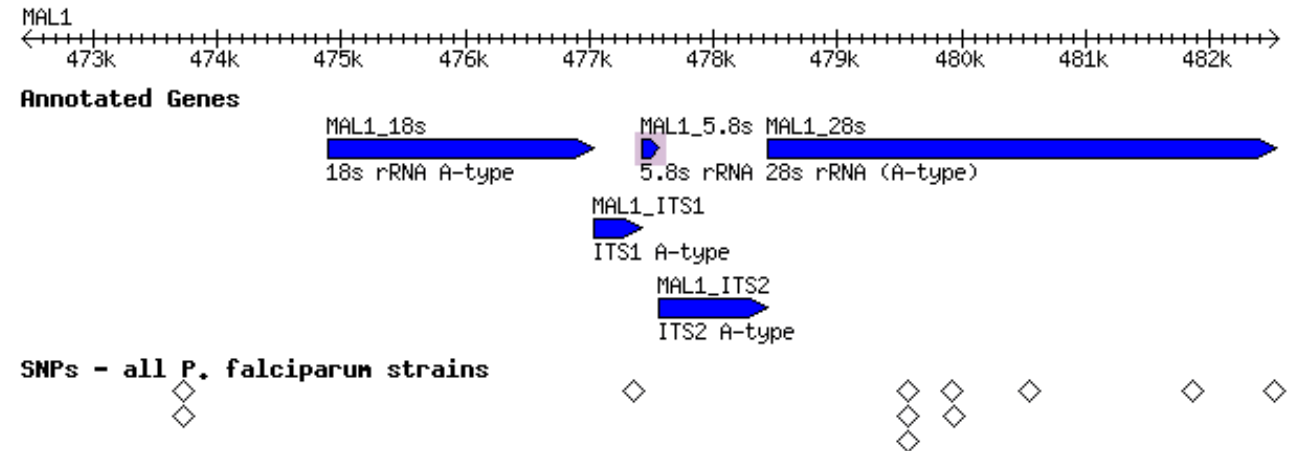

B

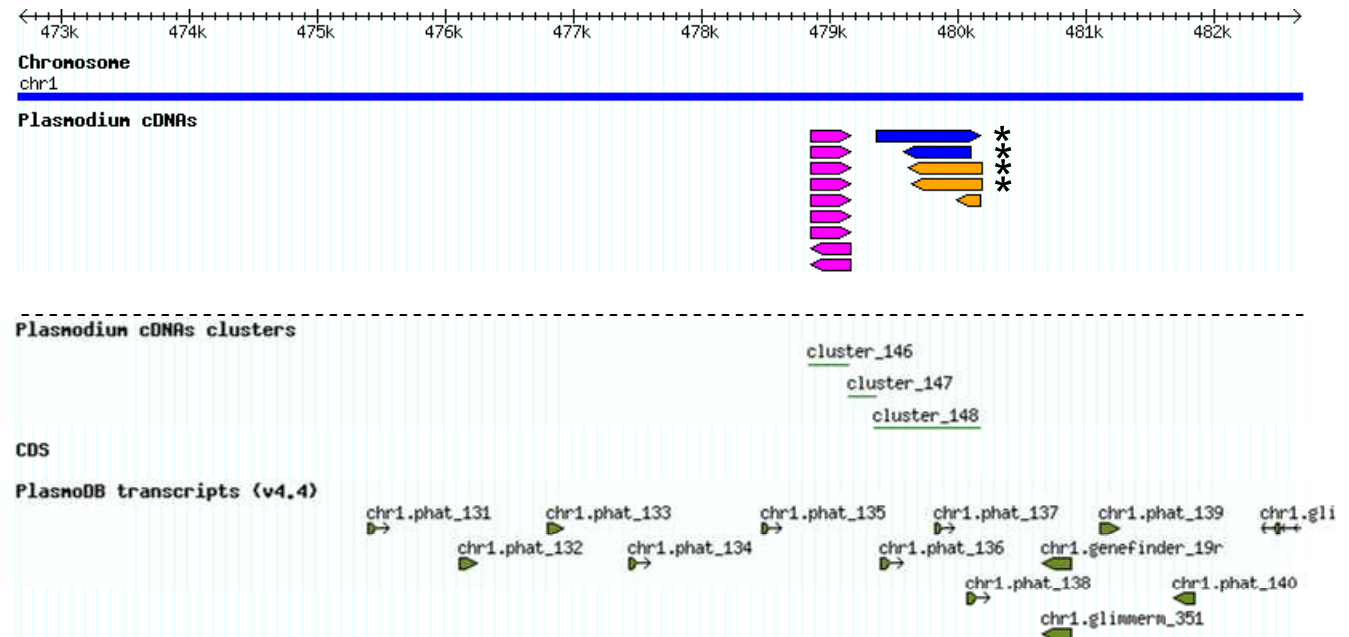

Supplement: Additional file 7 — Clustering of 626 FcB1-schizont-ESTs on the MAL1_28s gene of the chromosome 1 rRNA locus. A: view of the chromosome 1 locus (positions 473 k to 482 k) from PlasmoDB (version 4.4); B: view of the same locus on the Genoscope browser. Only the first, longest, 14 FcB1-ESTs are shown, the remaining 612 ESTs (corresponding to cluster_146 and cluster_147) are not shown here. The four largest ESTs (PU0AAA1YC08RM1, PU0AAA22YG18RM1, PU0AAA13YE12RM1, and PU0AAA44YM17RM1, indicated by *) are strictly specific to this chromosome 1 locus. Most of the smallest ESTs also clustered on homologous rRNA loci on chromosomes 5 and 7, MAL5_28s and MAL7_28s. (see Genoscope browser for details). [file 1471-2164-10-235-S7.pdf]

A

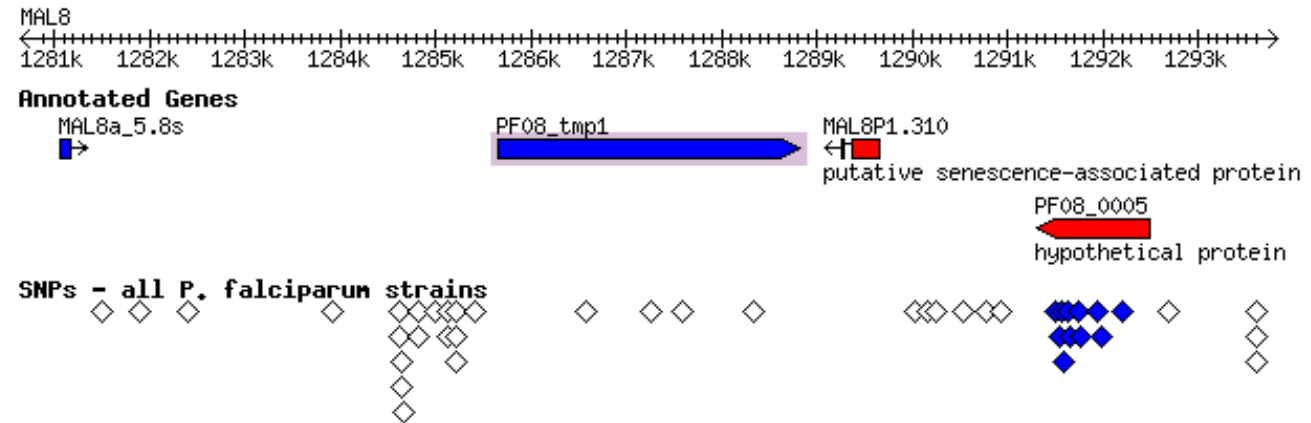

B

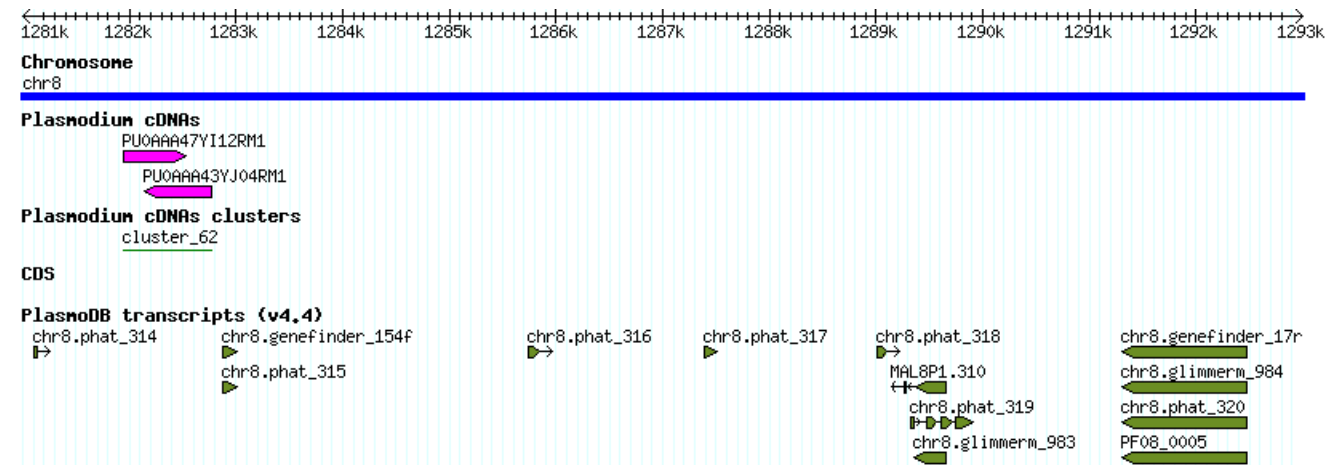

Supplement: Additional file 8 — FcB1-schizont-ESTs matching the atypical rRNA locus on chromosome 8, downstream of MAL8a_5.8s and upstream of PF08_tmp1. A: view of the chromosome 8 locus (positions 1281 k to 1293 k) from PlasmoDB (version 4.4); B. view of the same locus on the Genoscope browser. [file 1471-2164-10-235-S8.pdf]

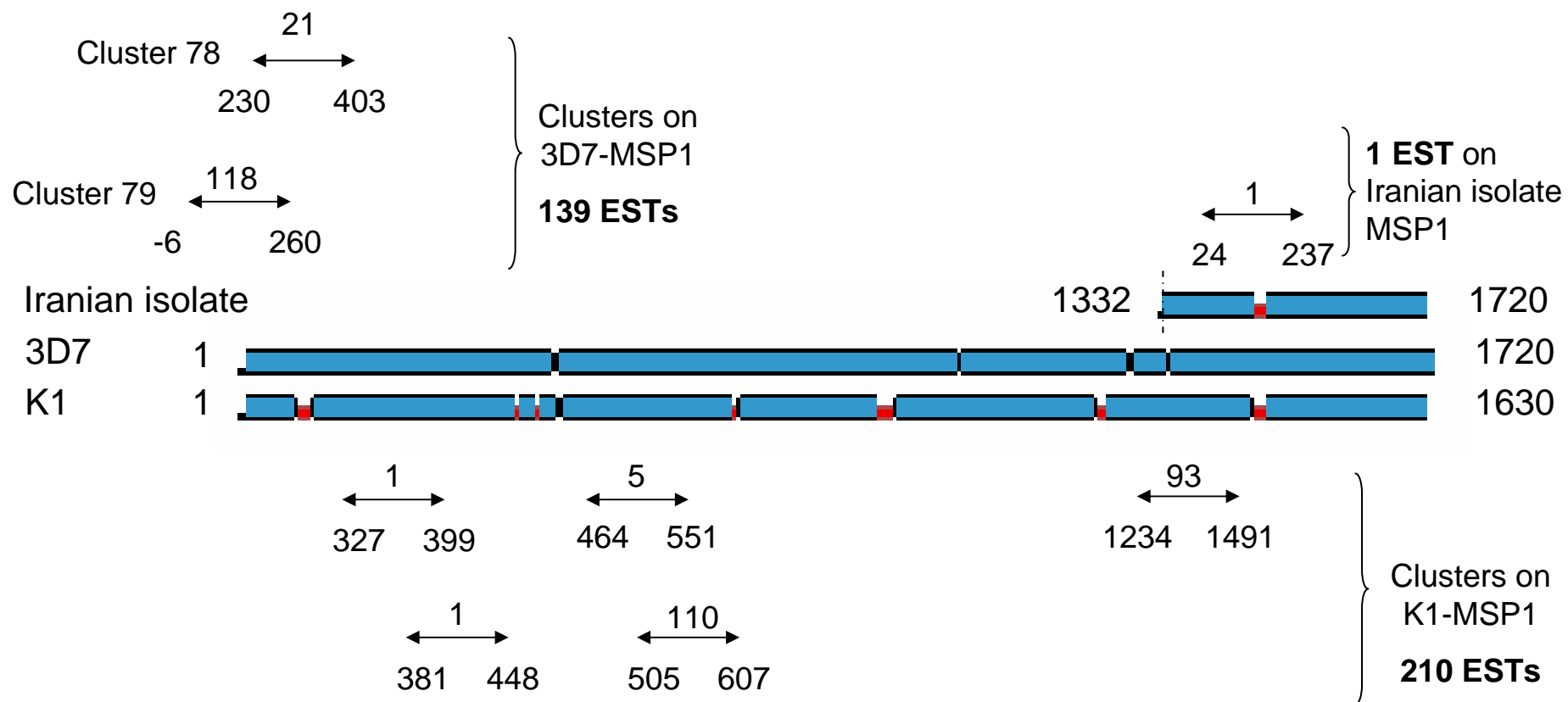

Supplement: Additional file 11 — Coverage of the MSP-1 gene by FcB1-schizont-ESTs. MSP1 protein sequences (1720 amino acids for the 3D7-type (downloaded from PlasmoDB), 1630 amino acids for the K1-type [UniProt:P04932], 1720 amino acids for the Iranian isolate [UniProt: A0SJF0/EMBL:DQ489588] were aligned and are represented schematically, red segments corresponding to gaps. The regions covered by the 139 ESTs matching 3D7-MSP1 are indicated above the alignment, the regions covered by 210 ESTs matching K1-MSP1 below. The EST matching the MSP1 variant [UniProt:A0SJF0] (Iranian isolate of K1-type) is also represented. Cluster_78 (corresponding to 21 ESTs matching amino-acid positions 230 to 403 of 3D7-MSP1) and cluster_79 (corresponding to 118 ESTs matching the N-terminal end of the protein to position 260) are indicated as arrows. The remaining 210 ESTs matching K1-MSP1 are represented as five groups with no cluster names. For each group, the number of ESTs is indicated above each arrow and the boundaries below each arrow. [file 1471-2164-10-235-S11.pdf]
