# Supplementary material for: A Plasmodium falciparum FcB1-schizont-EST collection providing clues to schizont specific gene structure and polymorphism
Source: BMC Genomics. 2009 May 19;10:235. doi: 10.1186/1471-2164-10-235 (PMC2695484; doi:10.1186/1471-2164-10-235)
Supplement: Additional file 5 — FcB1 versus 3D7 protein polymorphism. All 21 cases reported in Table 5 are illustrated. In each example, "FcB1" corresponds to the protein sequences deduced from a representative FcB1-schizont-EST and "3D7" to protein sequences deduced from the homologous 3D7 gene (EMBL/GenBank/DDBJ accession numbers indicated). Most sequence alignments were obtained using classical BLAST searches (default parameters) at NCBI . For Chr05_12, Chr12_15 and Chr13_12, sequence alignments were performed using the EMBOSS package available at EBI . [file 1471-2164-10-235-S5.pdf]

Chr01_03: PFA0215w

>ref|XP_001350975.1| hypothetical protein MAL1P1.70 [Plasmodium falciparum 3D7]
>emb|CAD49003.1| hypothetical protein [Plasmodium falciparum 3D7]
Length=2359 Score = 94.4 bits (233), Expect = 2e-18, Method: Composition-based stats. Identities = 141/153 (92%), Positives = 143/153 (93%), Gaps = 7/153 (4%)

```
FcB1   1     LFYKYKYSDNTHTKGSIYYIKKGSPRNSIKREDSNMYADQSVMAENIKKNYLNEGNQKDD  60
             LFYKYKYSDNTHTKGSIYYIKKGSPRNSIKREDSNMYADQSVMAENIKKNYLNEGNQKDD
3D7    1846  LFYKYKYSDNTHTKGSIYYIKKGSPRNSIKREDSNMYADQSVMAENIKKNYLNEGNQKDD  1905

FcB1   61    DNKNNYDDNKNNYDDKENNYDDNK-------NNYDDNKNNYDDKKNNYDDKKNNYDDNKN  113
             DNKNNYDD +NNYDDKENNYDD +       NNYDDNKNNYDDKKNNYDDKKNNYDDNKN
3D7   1906  DNKNNYDDKENNYDDKENNYDDKENNYDDNKNNYDDNKNNYDDKKNNYDDKKNNYDDNKN  1965

FcB1   114   NYDYNNNKNDDDDSINVSSSLKGIHKNTFDPFL  146
             NYDYNNNKNDDDDSINVSSSLKGIHKNTFDPF
3D7    1966  NYDYNNNKNDDDDSINVSSSLKGIHKNTFDPFF  1998
```

Chr04_01: PFD0185c

>ref|XP_001351350.1| hypothetical protein, conserved [Plasmodium falciparum 3D7]
>emb|CAD49130.1| hypothetical protein, conserved [Plasmodium falciparum 3D7]
Length=734 Score = 89.0 bits (219), Expect = 9e-17, Method: Composition-based stats. Identities = 105/128 (82%), Positives = 109/128 (85%), Gaps = 5/128 (3%)

```
FcB1   1    TILASIESIMKGIPDSNTLCEKILFKGIIIXKKNNSSNEIYSDCNPGYNYYTNNKYDNLY  60
            TILASIESIMKGIPDSNTLCEK   +     KKNNSSNEIYSDCNPGYNYYTNNKYDNLY
3D7    350  TILASIESIMKGIPDSNTLCEKNSVQRNNNIKKNNSSNEIYSDCNPGYNYYTNNKYDNLY  409

FcB1   61   SPNKVTSINNNKMDQ-----KNKNHKGNNKSSNNNNNNNSCSSSSNNSMSLNMKTLSSYT  115
            SPNKVTSINNNKMDQ     K  N   NN ++NNNNNNNSCSSSSNNSMSLNMKTLSSYT
3D7    410  SPNKVTSINNNKMDQKNKNHKGNNKSSNNNNNNNNNNNNSCSSSSNNSMSLNMKTLSSYT  469

FcB1   116  LKNKNTRG  123
            LKNKNT+G
3D7    470  LKNKNTQG  477
```

Chr05_06: PFE0250w

>ref|XP_001351609.1| hypothetical protein PFE0250w [Plasmodium falciparum 3D7]
>emb|CAD51416.1| hypothetical protein [Plasmodium falciparum 3D7]
Length=2754 Score = 263 bits (671), Expect = 6e-69, Method: Composition-based stats. Identities = 209/228 (91%), Positives = 210/228 (92%), Gaps = 12/228 (5%)

```
FcB1   1     HCGVEWGSQEYVLCNEKKNFERTNNNNGNNNGNNNDNNNDNNNDNNNGNNNDNNNYNNN-  59
             HCGVEWGSQEYVLCNEKKNFERTNNNN NNNGNNN NNN NNN NNN NNNDNNN NNN
3D7    1871  HCGVEWGSQEYVLCNEKKNFERTNNNNNNNNGNNNGNNNGNNNGNNNDNNNDNNNDNNNG  1930

FcB1   60    -----------GECRWDGEEKKNTIHKIRINKQLSIHNELYTNENVDKDINVFKQKFKHK  108
                        GECRWDGEEKKNTIHKIRINKQLSIHNELYTNENVDKDINVFKQKFKHK
3D7    1931  NNNDNNNYNNNGECRWDGEEKKNTIHKIRINKQLSIHNELYTNENVDKDINVFKQKFKHK  1990

FcB1   109   NIQHIIINGDIKKEDTFSLYFQVNTTYSGLHKCMMTMFCKKKNDEQMNKENKSSSNYLDI  168
             NIQHIIINGDIKKEDTFSLYFQVNTTYSGLHKCMMTMFCKKKNDEQMNKENKSSSNYLDI
3D7    1991  NIQHIIINGDIKKEDTFSLYFQVNTTYSGLHKCMMTMFCKKKNDEQMNKENKSSSNYLDI  2050

FcB1   169   LNQKKDGCYLFMRLMNVIPLIKLKIYSPLSIIHNDKYNYIYLSFENMC  216
             LNQKK+GCYLFMRLMNVIPLIKLKIYSPLSIIHNDKYNYIYLSFENMC
3D7    2051  LNQKKEGCYLFMRLMNVIPLIKLKIYSPLSIIHNDKYNYIYLSFENMC  2098
```

Chr05_12: PFE0655w

```
# 1: FcB1
# 2: 3D7
# Length: 2785
# Identity:     126/2785 ( 4.5%)
# Similarity:   134/2785 ( 4.8%)
# Gaps:        2629/2785 (94.4%)
# Score: 560.0


FcB1              60 ---KKKKKKIKLKLFINKDKNKNI------DKNIDKDKNIDKDKNIDKDK    101
                        |||||||||||||||||||||      ||||||||||||||||||||
3D7              181 --LKKKKKKIKLKLFINKDKNKNIDKNIDKDKNIDKDKNIDKDKNIDKDK    228

FcB1             102 NIDKDKNIDKENNELSSNNKTTNVEDMIIEDDNFMDNLNNNVDEYKKELY    151
                     ||||||||||||||||||||||||||||||||||||||||||||||||||
3D7              229 NIDKDKNIDKENNELSSNNKTTNVEDMIIEDDNFMDNLNNNVDEYKKELY    278

FcB1             152 FLNNYCLNFNKKKNYKGQKKK----------------------KKKKKX     178
                     |||||||||||||..|.:|||                      ..|||
3D7              279 FLNNYCLNFNKKKIIKAKKKKINFCEFSFTFSYNFKTLYTFFYTTKKKSI    328
```

Chr06_07: PFF0670w

>ref|XP_966125.1| hypothetical protein PFF0670w [Plasmodium falciparum 3D7]
>emb|CAG25377.1| hypothetical protein [Plasmodium falciparum 3D7]
Length=4095 Score = 242 bits (618), Expect = 8e-63, Method: Compositional matrix adjust.Identities = 149/171 (87%), Positives = 150/171 (87%), Gaps = 20/171 (11%)

```
FcB1   29     IKSMQNYMHHLNVTNDDLINNDNTNSKVLSNISQNHDSTKINSTIKIKESTSFEKETLNI  88
              IKSMQNYMHHLNVTNDDLINNDNTNSKVLSNISQNHDSTKINSTIKIKESTSFEKETLNI
3D7    1151   IKSMQNYMHHLNVTNDDLINNDNTNSKVLSNISQNHDSTKINSTIKIKESTSFEKETLNI  1210

FcB1   89     LTRSSKDDPNTCDNPEGVDININTQKKD--------------------NNNNNVHNNVHN  128
              LTRSSKDDPNTCDNPEGVDININTQKKD                    N +NNVHNNVHN
3D7    1211   LTRSSKDDPNTCDNPEGVDININTQKKDNNNNNNNNNNNNNNNNNVHNNVHNNVHNNVHN  1270

FcB1   129    NVHNNVHNNNFIKCIEKESNDGNTPHASPLIIKHNTDHMINFKCYSNDYII  179
              NVHNNVHNNNFIKCIEKESNDGNTPHASPLIIKHNTDHMINFKCYSNDYII
3D7    1271   NVHNNVHNNNFIKCIEKESNDGNTPHASPLIIKHNTDHMINFKCYSNDYII  1321
```

Chr06_11: PFF0765c

>ref|XP_966144.1| hypothetical protein PFF0765c [Plasmodium falciparum 3D7]
>emb|CAG25396.1| hypothetical protein [Plasmodium falciparum 3D7]
Length=1185 Score = 224 bits (572), Expect = 1e-57, Method: Composition-based stats. Identities = 154/163 (94%), Positives = 156/163 (95%), Gaps = 6/163 (3%)

```
FcB1   1     NLKYFSFTKLLKHIDKNNYFGDNQTSATVTFAQNFVNSISYISQENNEHEKNYKSYVVAE  60
             NLKYFSFTKLLKHIDKNNYFGDNQTSATVTFAQNFVNSISYISQENNEHEKNYKSYVVAE
3D7    731   NLKYFSFTKLLKHIDKNNYFGDNQTSATVTFAQNFVNSISYISQENNEHEKNYKSYVVAE  790


FcB1   61    NGVTDKCDVTNKYDVTNKYDVTNKYDVTNQ------YDVTNQYDVTNQYDVTNQYDVTNK  114
             NGVTDKCDVTNKYDVTNKYDVTN+YDVTNQ      YDVTNQYDVTNQYDVTNQYDVTNK
3D7    791   NGVTDKCDVTNKYDVTNKYDVTNQYDVTNQYDVTNKYDVTNQYDVTNQYDVTNQYDVTNK  850

FcB1   115   FLASDQHNTVAQYFKNYETPFDEIQKTKKGISDDADKKTFMSY  157
             +LASDQHNTVAQYFKNYETP DEIQKTKKGISDDADKKTFMSY
3D7    851   YLASDQHNTVAQYFKNYETPSDEIQKTKKGISDDADKKTFMSY  893
```

Chr07_06: MAL7P1.208

```
>gb|AAQ89710.1| rhoptry associated membrane antigen [Plasmodium falciparum]
Length=861 Score = 129 bits (323), Expect = 5e-31, Method: Compositional matrix
adjust.Identities = 104/195 (53%), Positives = 113/195 (57%), Gaps = 75/195 (38%)

FcB1   22   DEYEDN------------DESFLETEEYEDNEDEKYNKDEDD------------------  51
            DEYEDN             ESF+ET+EY+DNE+E+YNKDEDD
3D7    153  DEYEDNEDDKYNKDEDDYSESFIETDEYDDNEEEQYNKDEDDYADSFIETDHYENNDDKN  212

FcB1   52   ------------YAESFIETD-------------EYEEM--------------------E  66
                        Y  +F+ETD             EY E                    E
3D7    213  EEEEEYNDQDNDYGYNFLETDEYDDSEEYDYDDKEYGESFLEKEEGEEMKDEEMKDEEME  272

FcB1   67   DVEMKDEEMKDEEMKYDEMKNEEMKYDEMKDEVMKDEEMKDEQMKYEEFKNEEFKNEEFK  126
            DVEMKDEEMKDEEMKYDEMKNEEMKYDEMKDEVMKDEEMKDEQMKYEEFKNEEFKNEEFK
3D7    273  DVEMKDEEMKDEEMKYDEMKNEEMKYDEMKDEVMKDEEMKDEQMKYEEFKNEEFKNEEFK  332

FcB1   127  NEESKNEESKKKKKK  141
            NEESKNEESK ++ K
3D7    333  NEESKNEESKNEESK  347
```

Chr07_12: PF07_0111

```
>ref|XP_001349167.1| hypothetical protein PF07_0111 [Plasmodium falciparum 3D7]
>emb|CAD51013.1| hypothetical protein [Plasmodium falciparum 3D7]
Length=1162 Score = 329 bits (844), Expect = 3e-91, Method: Compositional matrix
adjust. Identities = 185/189 (97%), Positives = 185/189 (97%), Gaps = 4/189 (2%)

FcB1   20   NEKPNEESNEESSEKLNEEPNEKPN----EKPNEKPNKKTNTEPVPDLERAPFVGEENKI  75
            NEKPNEESNEESSEKLNEEPNEKPN    EKPNEKPNKKTNTEPVPDLERAPFVGEENKI
3D7    42   NEKPNEESNEESSEKLNEEPNEKPNEKPNEKPNEKPNKKTNTEPVPDLERAPFVGEENKI  101

FcB1   76   YVNEEDENFVNNYINENILNLKSVDNYLEKINNKIKDLDDNINDRIQKYILMKNEYEKMF  135
            YVNEEDENFVNNYINENILNLKSVDNYLEKINNKIKDLDDNINDRIQKYILMKNEYEKMF
3D7    102  YVNEEDENFVNNYINENILNLKSVDNYLEKINNKIKDLDDNINDRIQKYILMKNEYEKMF  161

FcB1   136  KRIKGRTKLINNIIDDIDKKTEKNQDVLINLCKDIKKLDTGKQNVTQTIILLKRIVILIT  195
            KRIKGRTKLINNIIDDIDKKTEKNQDVLINLCKDIKKLDTGKQNVTQTIILLKRIVILIT
3D7    162  KRIKGRTKLINNIIDDIDKKTEKNQDVLINLCKDIKKLDTGKQNVTQTIILLKRIVILIT  221

FcB1   196  AISKLKKKA  204
            AISKLKKKA
3D7    222  AISKLKKKA  230
```

Chr08_04: PF08_0109

```
>ref|XP_001349445.1| hypothetical protein PF08_0109 [Plasmodium falciparum 3D7]
>emb|CAD51294.1| hypothetical protein [Plasmodium falciparum 3D7]
Length=481 Score = 154 bits (389),  Expect = 1e-38, Method: Compositional matrix
adjust. Identities = 141/158 (89%), Positives = 143/158 (90%), Gaps = 14/158 (8%)

FcB1   26   DLPTIEEIENMKDIDNELKEALILSLREYTEKNKLENKDTT--------------SNNNN  71
            DLPTIEEIENMKDIDNELKEALILSLREYTEKNKLENKDTT              + +NN
3D7    246  DLPTIEEIENMKDIDNELKEALILSLREYTEKNKLENKDTTSNNNNNNNESNNNNNESNN  305

FcB1   72   NNNESNNNNNESNNNNNESNNNNNESNNNNNESNNNNNGNTCGNNNNNSLNFYKKDDNFA  131
            NNNESNNNNNESNNNNNESNNNNNESNNNNNESNNNNNGNTCGNNNNNSLNFYKKDDNFA
3D7    306  NNNESNNNNNESNNNNNESNNNNNESNNNNNESNNNNNGNTCGNNNNNSLNFYKKDDNFA  365

FcB1   132  LVNESYKNNFDKNDENKLETSKEKKENESYEKVFKIDK  169
            LVNESYKNNFDKNDENKLETSKEKKENESYEKVFKIDK
3D7    366  LVNESYKNNFDKNDENKLETSKEKKENESYEKVFKIDK  403
```

Chr10_06: PF10_0177

>ref|XP_001347462.1| erythrocyte membrane-associated antigen [Plasmodium falciparum 3D7]
>gb|AAN35375.1|AE014831_51 erythrocyte membrane-associated antigen [Plasmodium falciparum 3D7] Length=3165 Score = 146 bits (369), Expect = 8e-34, Method: Composition-based stats. Identities = 159/170 (93%), Positives = 162/170 (95%), Gaps = 8/170 (4%)

```
FcB1   44    EKNHKMDDSKDNIKRNINNVNYVKINHIDKEDKNKKVNYDDDKKVSDDDDKKVSDDDDNK  103
             EKNHKMDDSKDNIKRNINNVNYVKINHIDKEDKNKKVNYDDDKKVSDDDDKKVSDDDDNK
3D7    1326  EKNHKMDDSKDNIKRNINNVNYVKINHIDKEDKNKKVNYDDDKKVSDDDDKKVSDDDDNK  1385

FcB1   104   KVSDDDDKKVSDDDDKKVSDDDNKKVYYDDNKKVYYDDDKKINDDDNNNISKDIRYDEKN  163
             KVSDDDDKKVSDDDDKKVS+DDNKKVYYDD+KK+        NDDDNNNISKDIRYDEKN
3D7    1386  KVSDDDDKKVSDDDDKKVSNDDNKKVYYDDDKKI--------NDDDNNNISKDIRYDEKN  1437

FcB1   164   NFMEWMSHNKLMKNIISSYNEDVNIENHGLFNLLFDIYGFNKDIFKEYLV  213
             NFMEWMSHNKLMKNIISSYNEDVNIENHGLFNLLFDIYGFNKDIFKEYLV
3D7    1438  NFMEWMSHNKLMKNIISSYNEDVNIENHGLFNLLFDIYGFNKDIFKEYLV  1487
```

Chr10_07: PF10_0184

>ref|XP_001347469.1| hypothetical protein PF10_0184 [Plasmodium falciparum 3D7]
>gb|AAN35382.1|AE014832_4 hypothetical protein PF10_0184 [Plasmodium falciparum 3D7] Length=1928 Score = 145 bits (366), Expect = 7e-34, Method: Composition-based stats. Identities = 125/141 (88%), Positives = 130/141 (92%), Gaps = 1/141 (0%)

```
FcB1   1     KSQFFFPSFLSPPKKKKRKKK-SFILENEEFINEESSFDIQSNNINSHFIKNKNILNIKN  59
             K  +  P+ L P KKKK++KK SFILENEEFINEESSFDIQSNNINSHFIKNKNILNIKN
3D7    1245  KGMYLSPTSLPPQKKKKKEKKKSFILENEEFINEESSFDIQSNNINSHFIKNKNILNIKN  1304

FcB1   60    QTNDLENVKCYHHIKKQNNSKSQSSYFCSPAPSNSILRNNTESYFLKNDKQNDQKVKEDG  119
             QTNDLENVKCYHHIKKQNNSKSQSSYFCSPAPSNSILRNNTESYFLKNDKQNDQKVKEDG
3D7    1305  QTNDLENVKCYHHIKKQNNSKSQSSYFCSPAPSNSILRNNTESYFLKNDKQNDQKVKEDG  1364

FcB1   120   QTIKNQTTANNVQKKKKKKKV  140
             QTIKNQTTANNVQKK+ K  V
3D7    1365  QTIKNQTTANNVQKKRNKNYV  1385
```

Chr10_09: PF10_0213

>ref|XP_001352009.1| hypothetical protein, conserved [Plasmodium falciparum 3D7]
>emb|CAD51820.1| hypothetical protein, conserved [Plasmodium falciparum 3D7] Length=659 Score = 46.6 bits (109), Expect = 4e-04, Method: Composition-based stats. Identities = 33/77 (42%), Positives = 54/77 (70%), Gaps = 0/77 (0%)

```
FcB1   6    NKKNNQSGMNQQNVMNQQKVMNQPNAMNFPNNMNHPNNMNHPNNMNHLNNMNHPNNMNHL  65
            N KNN   +N  + +NQ   +NQ + +N  +N+NH +N+NH +N+NH +N+NH +N+NH
3D7    130  NDKNNVDNINHGDNINQVDNINQVDNINHGDNINHGDNINHGDNINHGDNINHGDNINHG  189

FcB1   66   NNMNHPNNINQPNLINY  82
            +N+NH +NIN  + IN+
3D7    190  DNINHGDNINHGDNINH  206
```

Chr10_15: PF10_0345

>emb|CAJ44190.1| merozoite surface protein 3 [Plasmodium falciparum]
>emb|CAJ44191.1| merozoite surface protein 3 [Plasmodium falciparum]
>emb|CAJ44192.1| merozoite surface protein 3 [Plasmodium falciparum]
Length=331 Score = 137 bits (345), Expect = 3e-31, Method: Composition-based stats. Identities = 184/189 (97%), Positives = 185/189 (97%), Gaps = 0/189 (0%)

```
FcB1   3    AVRAGTAASKVKKAFETASNAKKAAESALKTNETGERNSRNNFYTTKTKEYAGKVEKDYE  62
            A  A TAASKVKKAFETASNAKKAAESALKTNETGERNSRNNFYTTKTKEYAGKVEKDYE
3D7    111  AKEACTAASKVKKAFETASNAKKAAESALKTNETGERNSRNNFYTTKTKEYAGKVEKDYE  170

FcB1   63   RAKNAYQKANQAVLKAKEASSYDYILGWEFGGGVPEHKKEENMLSHLYVSSKDKENISKE  122
            RAKNAYQKANQAVLKAKEASSYDYILGWEFGGGVPEHKKEENMLSHLYVSSKDKENISKE
3D7    171  RAKNAYQKANQAVLKAKEASSYDYILGWEFGGGVPEHKKEENMLSHLYVSSKDKENISKE  230

FcB1   123  NDDVLDEKEEEAEETEEEELEEKNEEETESEISEDEEEEEEEEEEKKEEENGKKKEQEKEQ  182
            NDDVLDEKEEEAEETEEEELEEKNEEETESEISEDEEEEEEEEEE+KEEEN KKKEQEKEQ
3D7    231  NDDVLDEKEEEAEETEEEELEEKNEEETESEISEDEEEEEEEEEEEKEEENDKKKEQEKEQ  290

FcB1   183  SNENNDQKK  191
            SNENNDQKK
3D7    291  SNENNDQKK  299
```

Chr10_18: PF10_0351

>ref|XP_001347635.1| hypothetical protein PF10_0351 [Plasmodium falciparum 3D7] >gb|AAN35548.1|AE014834_45 hypothetical protein PF10_0351 [Plasmodium falciparum 3D7] Length=566 Score = 134 bits (337), Expect = 7e-33, Method: Compositional matrix adjust. Identities = 100/137 (72%), Positives = 106/137 (77%), Gaps = 21/137 (15%)

```
Query  6    QVPEEVAEELVEKVDEEVAEELVEKVDEKV----DPKVDEEVTEELIEKVDEEVTEELIE  61
            +VPEEVAEELVEKVDEEVAEELVEKVDEKV    D KVDEEVTEELIEKVDEEVTEELIE
Sbjct  153  KVPEEVAEELVEKVDEEVAEELVEKVDEKVAEEVDQKVDEEVTEELIEKVDEEVTEELIE  212

Query  62   KADEEVAEELIEKVDEEVAEELIEKVADELVEKVSEK-----------------NEEVEN  104
            K DEEVAEELIEKVDEEVAEELIEKVADEL+EKV E+                  EE+
Sbjct  213  KVDEEVAEELIEKVDEEVAEELIEKVADELIEKVDEEVAEELIEKVADELVEKVAEELVE  272

Query  105  KSETEIGEELTEKVDEK  121
            K + E+ EEL EKVDEK
Sbjct  273  KVDEEVAEELVEKVDEK  289
```

Chr12_05: PFL0465c

>ref|XP_001350502.1| Zinc finger transcription factor (krox1) [Plasmodium falciparum 3D7]
>gb|AAN36182.1|AE014845_37 Zinc finger transcription factor (krox1) [Plasmodium falciparum 3D7]Score = 132 bits (332), Expect = 3e-32, Method: Compositional matrix adjust. Identities = 67/68 (98%), Positives = 67/68 (98%), Gaps = 0/68 (0%)

```
FcB1   52   MSGIDSKNKRVICYVDENLNNNIYSESEKTFLKNNVDWNKIKLLNTSNIKPVVAGVEKKL  111
            MS IDSKNKRVICYVDENLNNNIYSESEKTFLKNNVDWNKIKLLNTSNIKPVVAGVEKKL
3D7    1    MSCIDSKNKRVICYVDENLNNNIYSESEKTFLKNNVDWNKIKLLNTSNIKPVVAGVEKKL  60

FcB1   112  VLDTSENK  119
            VLDTSENK
3D7    61   VLDTSENK  68
```

Chr12_15: PFL1385c

```
# 1: PFL1385c (3D7)
# 2: PU0AAA1YL15RM1_3 (FcB1)
# Length: 749
# Identity:     163/749 (21.8%)
# Similarity:   174/749 (23.2%)
# Gaps:         569/749 (76.0%)
# Score: 779.5
```

```
3D7              546 -YFKEEYYYDENDDMEVKVKKIGVTLKKFEPLKNGNVSETIKLIHLGNKD    594
                      .:...||.       |||||||||||||||||||||||||||||||||
FcB1               8 -IWVSNYYL-------VKVKKIGVTLKKFEPLKNGNVSETIKLIHLGNKD     50

3D7              595 KKHIEAINNDIQIIKQELQAIYNELMNYTNGNKNIQQIFQQNILENDVLN    644
                     ||||||||||||||||||||||||||||||||||||||||||||||||||
FcB1              51 KKHIEAINNDIQIIKQELQAIYNELMNYTNGNKNIQQIFQQNILENDVLN    100

3D7              645 QETEEEMEKQVEAITKQIEAEVDALAPKNKEEEEKEKEKEEKEKEEKEKE    694
                     ||||||||||||||||||||||||||||||||||||||| ||||||||||
FcB1             101 QETEEEMEKQVEAITKQIEAEVDALAPKNKEEEEKEKEK-EKEKEEKEKE    149

3D7              695 KEEKEKEEKEKEEKEKEEKEEEKKEKEEEQEEEEEEEIVPENLTTEESK    743
                     ::||||||||| ||||||||||||||||||:::::::.
FcB1             150 EKEKEKEEKEK-EKEKEEKEEEKKEKEEEQKKKKKKKK               186
```

Chr13_03: PF13_0053

```
>ref|XP_001349825.1| hypothetical protein PF13_0053 [Plasmodium falciparum 3D7]
>emb|CAD52232.1| hypothetical protein [Plasmodium falciparum 3D7]
Length=1672 Score = 156 bits (394), Expect = 5e-37, Method: Composition-based
stats. Identities = 109/116 (93%), Positives = 110/116 (94%), Gaps = 6/116 (5%)

FcB1    1      KNVFKRSGNMQYDINEQDNTNEQDNINKQDNINKQDNINKQDNTN------EQDNTNEQN  54
               KNVFKR+GNMQYDINEQDNTNEQDNINKQDNINKQDNINKQDNTN      EQDNTNEQN
3D7     1233   KNVFKRNGNMQYDINEQDNTNEQDNINKQDNINKQDNINKQDNTNKQDNTNEQDNTNEQN  1292

FcB1    55     SVNEKDIINSQDNLTSRENIYLYNKPINFKYQNKHTEECIRFLEYLTNKLIEENIL  110
               SVNEKDIINSQDNLTSRENIYLYNKPINFKYQNKHTEECIRFLEYLTNKLIEENIL
3D7     1293   SVNEKDIINSQDNLTSRENIYLYNKPINFKYQNKHTEECIRFLEYLTNKLIEENIL  1348
```

Chr13_12: MAL13P1.158

```
# 1: PU0AAA8YO05RM1_6 (FcB1)
# 2: MAL13P1.158 (3D7)
# Length: 697
# Identity:      88/697 (12.6%)
# Similarity:   101/697 (14.5%)
# Gaps:         568/697 (81.5%)
# Score: 372.5


FcB1              37 ---------------KKKWISKKMWIKKKI-----------------VDT     66
                                    .:....||::||||:                 |||
3D7              141 LMKIWKKGFYRTNIASQNNKKKKLYIKKKVDKQKNVDTPKNVDTPKNVDT    190

FcB1              67 PKNVDTPKNVDTPKNVDTKKHLDTYNNICINKNTLRYILFSLYNNKCRGE    116
                     ||||||||||||||||||||||||||||||||||||||||||||||||||
3D7              191 PKNVDTPKNVDTPKNVDTKKHLDTYNNICINKNTLRYILFSLYNNKCRGE    240

FcB1             117 CLKDDYKINLIKSKKKKKK-----KKKV                          139
                     |||||||||||||...|.:     ..|:
3D7              241 CLKDDYKINLIKSIINKWEIIPFINDKLLLILMLENLQFLYSNNYKLVSV    290
```

Chr13_22: PF13_0245

```
>ref|XP_001350179.1| hypothetical protein PF13_0245 [Plasmodium falciparum 3D7]
>emb|CAD52588.1| hypothetical protein [Plasmodium falciparum 3D7]
Length=595 Score =  163 bits (412),  Expect = 4e-39, Method: Compositional matrix
adjust. Identities = 78/81 (96%), Positives = 79/81 (97%), Gaps = 0/81 (0%)

FcB1   1    LNYNHFFTYPILKNEKIKNIYPIVPYISVWENKYIQGIMDIESDTEQITPSGNQTNKSND  60
            LNYNHFF +PILKNEKIKNIYPIVPYISVWENKYIQGIMDIESDTEQIT SGNQTNKSND
3D7    259  LNYNHFFRHPILKNEKIKNIYPIVPYISVWENKYIQGIMDIESDTEQITTSGNQTNKSND  318

FcB1   61   NKFYGLLHLIQKSRDKHIYSL  81
            NKFYGLLHLIQKSRDKHIYSL
3D7    319  NKFYGLLHLIQKSRDKHIYSL  339
```

Chr14_08: PF14_0175

```
>ref|XP_001348348.1| hypothetical protein PF14_0175 [Plasmodium falciparum 3D7]
>gb|AAN36787.1|AE014818_52 hypothetical protein [Plasmodium falciparum 3D7]
Length=4662 Score = 43.9 bits (102),  Expect = 3e-05, Method: Compositional matrix
adjust. Identities = 25/73 (34%), Positives = 46/73 (63%), Gaps = 0/73 (0%)

FcB1   35    ATNESTTNKSTTNESTTNKSTTNKPTTNKPTTNKPTTNKSTTNKSTTNKPTTNKSTTNKS  94
             + N+   N  + N+S TN    NKP+T+  + N+P+TN  + NKS+TN  + N+++ N
3D7    4002  SMNQPNINNFSMNQSNTNNFNMNKPSTSSFSMNQPSTNNFSMNKSSTNNFSINQTSANNF  4061

FcB1   95    TTNKPTTNKSTIN  107
             + ++P+TN  ++N
3D7    4062  SMHQPSTNNFSMN  4074
```

Chr14_29: PF14_0486

```
>ref|XP_001348660.1| elongation factor 2 [Plasmodium falciparum 3D7]
>gb|AAN37099.1|AE014824_18 elongation factor 2 [Plasmodium falciparum 3D7]
Length=832 Score =  170 bits (431),  Expect = 1e-43, Method: Compositional matrix
adjust. Identities = 79/81 (97%), Positives = 79/81 (97%), Gaps = 0/81 (0%)

FcB1   6    GTPLLKIQSHFLVSESFGFTSALRAATSGQAFPQCVFDHWSVLYDDPFDSNKNSYKIIMN  65
            GTPLLKIQSH  VSESFGFTSALRAATSGQAFPQCVFDHWSVLYDDPFDSNKNSYKIIMN
3D7    752  GTPLLKIQSHLPVSESFGFTSALRAATSGQAFPQCVFDHWSVLYDDPFDSNKNSYKIIMN  811

FcB1   66   IRERKGIKVEMPQLDQYLDKL  86
            IRERKGIKVEMPQLDQYLDKL
3D7    812  IRERKGIKVEMPQLDQYLDKL  832
```
