# Supplementary material for: A Plasmodium falciparum FcB1-schizont-EST collection providing clues to schizont specific gene structure and polymorphism
Source: BMC Genomics. 2009 May 19;10:235. doi: 10.1186/1471-2164-10-235 (PMC2695484; doi:10.1186/1471-2164-10-235)
Supplement: Additional file 6 — List of 26 FcB1-schizont-ESTs representative of the 9983 FcB1-ESTs matching rRNA loci on chromosomes 5 and 7. To limit this redundancy in the public databases, 26 sequences corresponding to the longest ESTs on each cluster were selected to be deposited in EMBL. (a) PU number of the EST; (b) size in bp; (c) corresponding rRNA element (5.8s, 18s, 28s or ITS1, internal transcribed spacer 1); (d, e, f) position of Hit on chromosome 5 (d), chromosome 7 (e) and chromosome 1 (f), respectively. [file 1471-2164-10-235-S6.pdf]

Name of EST (a)	Size (b)	rRNA type (c)	Hit on chromosome 5 (d)	Hit on chromosome 7 (e)	Hit on chromosome 1 (f)
PU0AAA10YH10RM1	655_bp	28s rRNA	chr5:1294719..1295373 (655 bp)	chr7:1144262..1144916 (655 bp)	
PU0AAA11YN02RM1	660_bp	18s rRNA	chr5:1289594..1290253 (660 bp)	chr7:1139139..1139798 (660 bp)	
PU0AAA1YB12RM1	628_bp	28s rRNA		chr7:1144975..1145602 (628 bp)	
PU0AAA32YB23RM1	305_bp	28s rRNA	chr5:1294127..1294431 (305 bp)	chr7:1143670..1143974 (305 bp)	
PU0AAA44YE03RM1	285_bp	18s rRNA	chr5:1290768..1291052 (285 bp)	chr7:1140310..1140594 (285 bp)	
PU0AAA47YN24RM1	299_bp	18s rRNA	chr5:1290478..1290776 (299 bp)	chr7:1140020..1140318 (299 bp)	
PU0AAA4YJ10RM1	320_bp	28s rRNA		chr7:1142334..1142646 (313 bp)	chr1:478841..479160 (320 bp)
PU0AAA50YB24RM1	363_bp	28s rRNA	chr5:1292403..1292765 (363 bp)		
PU0AAA52YG20RM1	205_bp	28s rRNA	chr5:1293103..1293307 (205 bp)		
PU0AAA53YK03RM1	230_bp	28s rRNA	chr5:1294279..1294508 (230 bp)	chr7:1143822..1144051 (230 bp)	
PU0AAA54YH19RM1	162_bp	5.8s rRNA	chr5:1292046..1292207 (162 bp)	chr7:1141592..1141753 (162 bp)	
PU0AAA55YC03RM1	364_bp	28s rRNA		chr7:1141946..1142309 (364 bp)	
PU0AAA56YL09RM1	148_bp	5.8s rRNA	chr5:1292043..1292190 (148 bp)	chr7:1141589..1141736 (148 bp)	
PU0AAA58YC12RM1	299_bp	28s rRNA		chr7:1142357..1142648 (292 bp)	chr1:478864..479162 (299 bp)
PU0AAA60YF24RM1	232_bp	28s rRNA	chr5:1292813..1293044 (232 bp)	chr7:1142357..1142588 (232 bp)	
PU0AAA6YJ14RM1	275_bp	18s + ITS1	chr5:1291519..1291793 (275 bp)		
PU0AAA8YN12RM1	396_bp	28s rRNA		chr7:1143653..1144048 (396 bp)	
PU0AAA8YO07RM1	600_bp	28s rRNA	chr5:1293292..1293891 (600 bp)	chr7:1142835..1143433 (599 bp)	
PU0AAA8YP01RM1	363_bp	28s rRNA		chr7:1141947..1142309 (363 bp)	
PU0AAA8YP06RM1	481_bp	18s rRNA	chr5:1289594..1290074 (481 bp)	chr7:1139139..1139619 (481 bp)	
PU0AAA9YA22RM1	440_bp	28s rRNA		chr7:1141958..1142397 (440 bp)	
PU0AAA9YD17RM1	168_bp	28s rRNA		chr7:1145388..1145555 (168 bp)	
PU0AAA9YI19RM1	445_bp	28s rRNA	chr5:1295369..1295813 (445 bp)	chr7:1144912..1145356 (445 bp)	
PU0AAA9YK09RM1	632_bp	18s rRNA	chr5:1289705..1290336 (632 bp)	chr7:1139250..1139880 (631 bp)	
PU0AAA9YL11RM1	146_bp	5.8s rRNA	chr5:1292043..1292188 (146 bp)	chr7:1141589..1141734 (146 bp)	
PU0AAA9YP23RM1	285_bp	18s rRNA	chr5:1290770..1291054 (285 bp)	chr7:1140312..1140596 (285 bp)	
