# Supplementary material for: A Plasmodium falciparum FcB1-schizont-EST collection providing clues to schizont specific gene structure and polymorphism
Source: BMC Genomics. 2009 May 19;10:235. doi: 10.1186/1471-2164-10-235 (PMC2695484; doi:10.1186/1471-2164-10-235)
Supplement: Additional file 9 — FcB1-schizont-ESTs matching sub-telomeric regions of chromosomes. A: schematic representation of P. falciparum sub-telomeric regions and consensus sequences reported for each structure, compiled from [29] and [30]. B: alignment of representative ESTs for Chr05_01 = Chr13_01 (cluster_304 and cluster_188, PU0AAA56YB23RM1), Chr10_01 (cluster_98, PU0AAA22YJ11RM1), Chr08_01 (cluster_64, PU0AAA57YH13RM2) and Chr07_15 (cluster_48, PU0AAA27YL11RM1) on the end of chromosome 3 (EMBL AL034560), shown as a typical example, These FcB1-schizont-ESTs are localized in TARE regions, between telomere and R-CG7 segments [31]. [file 1471-2164-10-235-S9.pdf]

A.

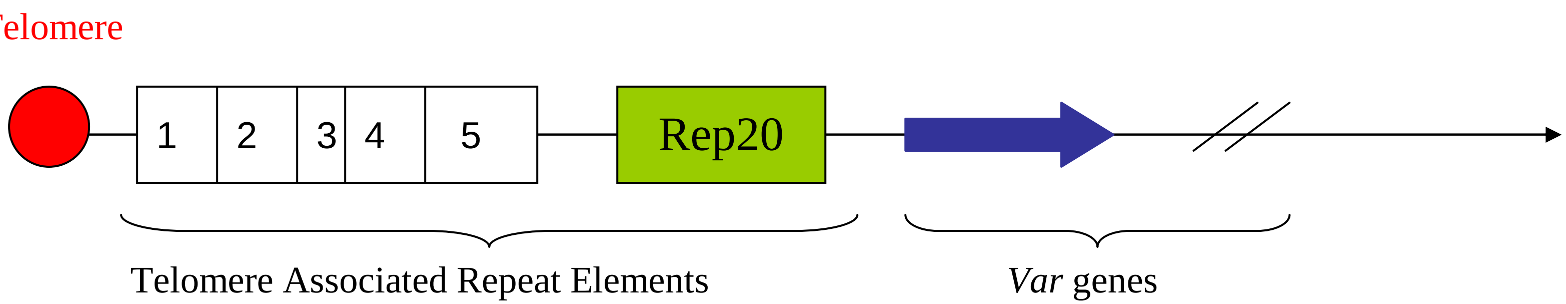

Structure	length	Consensus sequence
Telomere	~1kb	GGGTT(T/C)A
TARE1	0.9 to 1.9kb	Complex tandem repeats
TARE2	1.6 kb	135bp degenerate sequence, repeated 12 times, interspersed by two distinct 21 bp sequences
TARE3	0.7 kb	Three to four consecutive 0.7 kb elements
TARE4	0.7 to 2 kb	Highly degenerate short repeat and an interspersed non-repetitive sequence of 230 bp
TARE5	1.4 to 2 kb	Moderately degenerate tandem repeats of 12bp, ACTAACA(T/A)(C/G)A(T/C) (T/C)
TARE6 = Rep20	8.4 to 21 kb	Contains a degenerate 21bp sequence, TAAGACCTA & TTAGTA/T

B.

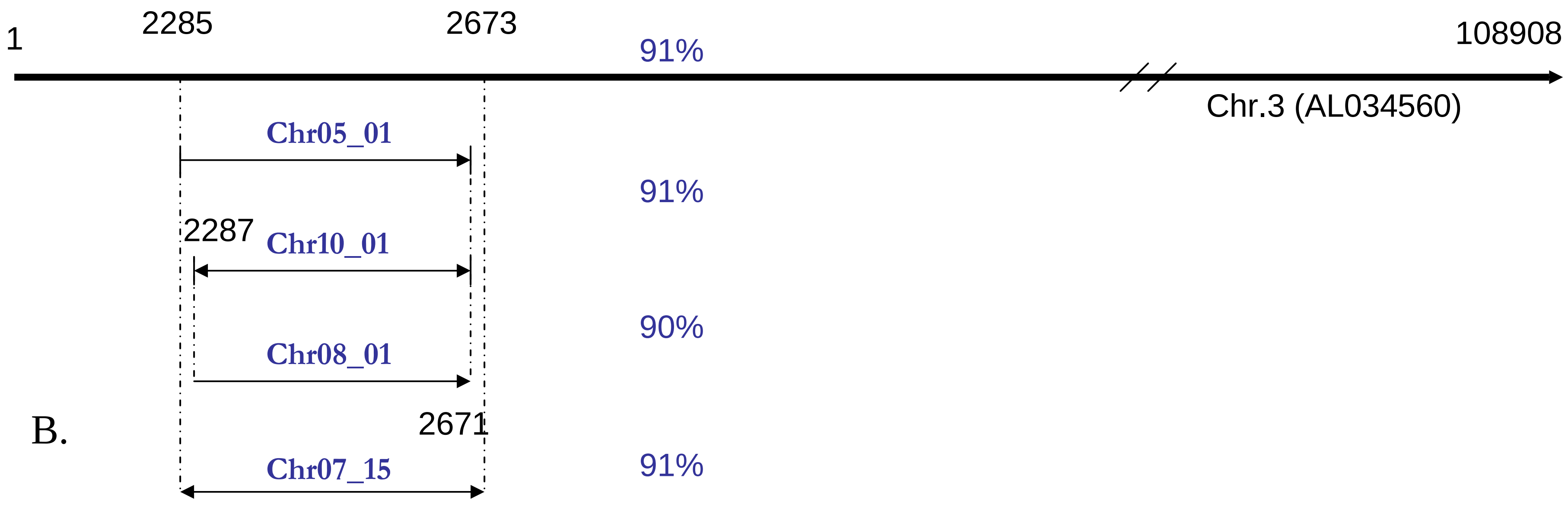

EMBL AL034560, chromosome 3, 108908 bp : annotations	
1…273	Telomeric repeat region
4530…6818	R-CG7 = TARE3
8638…10564	rep11
10610…30944	rep20 = TARE6
join(33641..38959, 39848..41158)	MAL3P8.1 = PFC0005w *var* gene (PfEMP1)
