# Supplementary material for: A Plasmodium falciparum FcB1-schizont-EST collection providing clues to schizont specific gene structure and polymorphism
Source: BMC Genomics. 2009 May 19;10:235. doi: 10.1186/1471-2164-10-235 (PMC2695484; doi:10.1186/1471-2164-10-235)
Supplement: Additional file 10 — FcB1-schizont-ESTs matching the template boundary element (TBE) and template regions of P. falciparum telomerase RNA. The sequence of P. falciparum telomerase RNA (Pf TR telomerase) was aligned with sequences of the five FcB1-schizont-ESTs (PU0AAA accessions numbers indicated) by using ClustalW showing extensive conservation. Template boundary element (TBE) and template regions, both located at the 5'-end of the P. falciparum telomerase RNA, are as defined by [32]. [file 1471-2164-10-235-S10.pdf]

```
PU0AAA36YI02RM1                     --------------------ACATTTTATTTCTTTTTTTTTTTTTTT-CT 29
PU0AAA54YB05RM1                     --------------------ACATTTTATTTCTTTTTTTTTTTTTTT-CT 29
PU0AAA54YL13RM1                     --------------------ACATTTTATTTCTTTTTTTTTTTTTTTTCT 30
PU0AAA36YD05RM1                     --------------------ACATTTTATTTCTTTTTTTTTTTTTTTTCT 30
PU0AAA35YH12RM1                     --------------------ACATTTTATTTCTTTTTTTTTTTTTTTTTT 30
Pf TR-telomerase                    GTAGTTCTATTTTTCTTATTTCATTTTATTTCTTTTTTTTTTTTTTTTTT 650
                                                         **************************  *

PU0AAA36YI02RM1                     TT----TCCCCATTTTTTCCCAAACATTCTTATTTTGGAGTGTTTGAAAA 75
PU0AAA54YB05RM1                     TT----TCCCCATTTTTTCCCAAACATTCTTATTTTGGAGTGTTTGAAAA 75
PU0AAA54YL13RM1                     TT----TCCCCATTTTTTCCCAAACATTCTTATTTTGGAGTGTTTGAAAA 76
PU0AAA36YD05RM1                     TT----TCCCCATTTTTTCCCAAACATTCTTATTTTGGAGCGTTTGAAAA 76
PU0AAA35YH12RM1                     TT--TTTCCCCATTTTTTCCCAAACATTCTTATTTTGGAGTGTTTGAAAA 78
Pf TR-telomerase                    TTCTTTTCCCCATTTTTTCCCAAACATTCTTATTTTGGAGTGTTTGAAAA 700
                                    **    ********************************** *********

PU0AAA36YI02RM1                     AAAGTGGAGGAAAAAAAAAAAAAAAAA---TATAAGAAAGAAAAAGAAAA 122
PU0AAA54YB05RM1                     AAAGTGGAGGAAAAAAAAAAAAAAA-----TATAAGAAAGAAAAAGAAAA 120
PU0AAA54YL13RM1                     AAAGTGGAGGAAAAAAAAAAAAAAAA----TATAAGAAAGAAAAAGAAAA 122
PU0AAA36YD05RM1                     AAAGTGGAGGAAAAAAAAAAAAAAAA----TATAAGAAAGAAAAAGAAAA 122
PU0AAA35YH12RM1                     AAAGTGGAGGAAAAAAAAAAAAAAAA---ATATAAGAAAGAAAAAGAAAA 125
Pf TR-telomerase                    AAAGTGGAGGAAAAAAAAAAAAAAAAAAAATATAAGAAAGAAAAAGAAAA 750
                                    *************************     ********************

                                                                            TBE
                                                                       <<<<<    >>>>>_
PU0AAA36YI02RM1                     AAGAAAAAATGATCATCGAAATCCTTAGAAAGCATGGGAAGAAATTCTCA 172
PU0AAA54YB05RM1                     AAGAAAAAATGATCATCGAAATCCTTAGAAAGCATGGGAAGAAATTCTCA 170
PU0AAA54YL13RM1                     AAGAAAAAATGATCATCGAAATCCTTAGAAAGCATGGGAAGAAATTCTCA 172
PU0AAA36YD05RM1                     AAGAAAAAATGATCATCGAAATCCTTAGAAAGCATGGGAAGAAATTCTCA 172
PU0AAA35YH12RM1                     AAGAAAAAATGATCATCGAAATCCTTAAAAAGCATGGGAAGAAATTCTCA 175
Pf TR-telomerase                    AAGAAAAAATGATCATCGAAATCCTTAGAAAGCATGGGAAGAAATTCTCA 800
                                    *************************** **********************

                                    Template
                                    __________
PU0AAA36YI02RM1                     CCCTGAACCCATAAAGTAGCTGATTTTTTGCTCATAAGT----------- 211
PU0AAA54YB05RM1                     CCCTGAACCCATAAAGTAGCTGATTTTTTGCTCATAAGT----------- 209
PU0AAA54YL13RM1                     CCCTGAACCCATAAAGTAGCTGATTTTTTGCTCATAAGT----------- 211
PU0AAA36YD05RM1                     CCCTGAACCCATAAAGTAGCTGATTTTTTGCTCATAAGT----------- 211
PU0AAA35YH12RM1                     CCCTGAACCCATAAAGTAGCTGATTTTTTGCTCATAAGT----------- 214
Pf TR-telomerase                    CCCTGAACCCATAAAGTAGCTGATTTTTTGCTCATAAGTACCTGAATTAT 850
                                    ***************************************
```
